# Targeting TCTP sensitizes tumor to T cell-mediated therapy by reversing immune-refractory phenotypes

**DOI:** 10.1038/s41467-022-29611-y

**Published:** 2022-04-19

**Authors:** Hyo-Jung Lee, Kwon-Ho Song, Se Jin Oh, Suyeon Kim, Eunho Cho, Jungwon Kim, Yun gyu Park, Kyung-Mi Lee, Cassian Yee, Seung-Hwa Song, Suhwan Chang, Jungmin Choi, Sang Taek Jung, Tae Woo Kim

**Affiliations:** 1 Department of Biochemistry and Molecular Biology, Korea university College of Medicine, Seoul, 02841 Republic of Korea; 2 Department of Biomedical Science, Korea university College of Medicine, Seoul, 02841 Republic of Korea; 3 Research Institute of Biomedical Engineering and Department of Medicine, Catholic University of Daegu School of Medicine, Daegu, Republic of Korea; 4 BK21 Graduate Program, Korea university College of Medicine, Seoul, 02841 Republic of Korea; 5 Department of Melanoma Medical Oncology and Immunology, U.T. MD Anderson Cancer Center, Houston, TX USA; 6 Clinical Research Division, Fred Hutchinson Cancer Research Center, Seattle, WA USA; 7 Department of Biomedical Sciences, University of Ulsan College of Medicine, Asan Medical Center, Seoul, 05505 Republic of Korea; 8 Department of Physiology, University of Ulsan College of Medicine, Asan Medical Center, Seoul, 05505 Republic of Korea; 9 NEX-I Inc., Seoul, 05854 Republic of Korea

**Keywords:** Cancer immunotherapy, Immunoediting

## Abstract

Immunotherapy has emerged as a powerful approach to cancer treatment. However, immunotherapeutic resistance limits its clinical application. Therefore, identifying immune-resistant factors, which can be targeted by clinically available drugs and it also can be a companion diagnostic marker, is needed to develop combination strategies. Here, using the transcriptome data of patients, and immune-refractory tumor models, we identify TCTP as an immune-resistance factor that correlates with clinical outcome of anti-PD-L1 therapy and confers immune-refractory phenotypes, decreased T cell trafficking to the tumor and resistance to cytotoxic T lymphocyte-mediated tumor cell killing. Mechanistically, TCTP activates the EGFR-AKT-MCL-1/CXCL10 pathway by phosphorylation-dependent interaction with Na, K ATPase. Furthermore, treatment with dihydroartenimsinin, the most effective agent impending the TCTP-mediated-refractoriness, synergizes with T cell-mediated therapy to control immune-refractory tumors. Thus, our findings suggest a role of TCTP in promoting immune-refractoriness, thereby encouraging a rationale for combination therapies to enhance the efficacy of T cell-mediated therapy.

## Abstract (web summary)

Translationally controlled tumor protein (TCTP) regulates several cellular processes, including apoptosis, and is overexpressed in several cancer types. Here, the authors report that high levels of TCTP are associated with poor response to anti-PD-L1 and that TCTP targeting increases the efficacy of T cell-mediated anti-tumor therapy.

## Introduction

Cancer immunotherapy is a strategy to treat tumors by leveraging the cytotoxic potential of the human immune system^1^. Particularly, T cell-mediated therapeutic methods such as adoptive T cell therapy (ACT) using tumor-specific T cells and CAR T cells, and immune checkpoint blockade (ICB) have achieved tremendous progress in the field of cancer immunotherapy. However, the presence of immunotherapeutic resistance limits the clinical application of T cell-mediated therapy^2^. Resistance is a result of complex and constantly evolving interactions between tumor cells and the immune system. Among the diverse causes, the cancer immunoediting theory that drives the adaptation of tumor cells to the host immune system has attracted attention as it can explain the emergence of resistance to anti-tumor immunity^3^. In this process, cytotoxic T lymphocyte (CTL)-mediated immune selection triggers the evolution of tumors toward better survival advantage and immune-refractory phenotypes to T cell-mediated therapy^4^. Interestingly, these immune-refractory tumors not only exhibit resistance to CTL-mediated killing but also restrict anti-cancer immunity^5^. Thus, understanding the pathways that regulate the immune-refractory phenotypes could present targets to potentiate the efficacy of T cell-mediated therapies.

For a successful anti-cancer immune response, a series of step-wise events, the cancer immunity cycle, must be initiated and allowed to proceed and expand iteratively^6^. Therefore, reinforcing various steps of the cancer immunity cycle, such as tumor immunogenicity or T cell priming, and the cytotoxic capacity of T cells are proposed as combinational strategies with T cell-mediated therapy^7^. However, disruption of multiple steps of the cycle obstructs the anti-cancer immunity, thereby leading to immunotherapy failure^8,9^. Especially, the T cells trafficking into tumor and their subsequent eradication of tumor cells are two rate-limiting steps determine the anti-cancer immune response^10^. Decreased T cell trafficking to tumors appears in low immunotherapy responses compared to T cell-recruited tumors in patients^10,11^. Besides, even if T cells infiltrate a tumor, the intrinsic resistance of tumor cells to CTL-mediated killing could be another obstacle^12–14^. Thus, controlling both T cell trafficking to the tumor and the anti-apoptotic properties of tumor cells to CTLs is needed to improve the effectiveness of CTL-mediated therapy.

Accumulating evidence indicates that the role of oncogenic pathways in tumor cells is not always confined to tumorigenesis, and can be extended to interfering anti-tumor immune responses^15–17^. For instance, the activation of PI3K-AKT signaling in tumors not only inhibited the CTL-mediated tumor cells lysis also decreased T cell trafficking to the tumor^17^. Notably, targeting AKT signaling rendered tumors susceptible to CTL-mediated killing^18,19^, and increased trafficking of tumor-reactive T cells via the amplification of anti-tumor immunity^5^. These results indicate that targeting oncogenic pathways that confers immune-refractory phenotypes could be combined with T cell-mediated therapy as a potential strategy to overcome resistance. Thus, elucidating resistant pathways that can be targeted by clinically available drugs is needed to develop tailored combination strategies with CTL-mediated therapy.

Translationally controlled tumor protein (TCTP) is multifunctional protein ubiquitously expressed in eukaryotes and highly conserved across a wide range of species^20–22^. TCTP regulates cell cycle progression, growth, anti-apoptosis, and malignant transformation by interacting with various proteins^23–25^. In cancer, TCTP is overexpressed in various types of cancer cells and is associated with poor prognosis^26–28^. Interestingly, TCTP not only induces multi-malignancy but also confers chemo- and irradiation-resistance in various types of cancer by regulating oncogenic signaling such as the EGFR-AKT pathway^29–32^. Thus, clinically applicable agents that could target TCTP, such as dihydroartemisinin (DHA), rapamycin, sertraline, and thioridazine, were suggested to regress cancer^33–36^. Although the importance of TCTP as a therapeutic target continues to grow, the potential relationship between TCTP and immunotherapeutic resistance and the possibility of TCTP as a actionable target for combination strategies with immunotherapy have not been explored.

Here, we show TCTP as an immune-resistance factor that confers immune-refractory phenotypes, decreased T cell infiltration to the tumor and resistance of tumor cells to CTL-mediated killing. Notably, the TCTP expression status serve as a companion diagnostic marker that is associated with poor response to ICB and ACT. Mechanistically, TCTP activates the EGFR-AKT pathway by phosphorylation-dependent binding with Na, K ATPase α1, thereby regulating CXCL10 and MCL-1. Furthermore, by screening several TCTP-targeting agents, we identify DHA as the most effective agent in enhancing the CTL-mediated cytotoxicity of tumor cells. TCTP inhibition with DHA sensitizes tumors to T cell-mediated therapy, including anti-PD-L1 and ACT therapy, by increasing tumor-reactive T cells in the tumor and the CTL-mediated lysis of tumor cells. Thus, we provide a proof of principle that targeting TCTP signaling is an appealing therapeutic strategy to combine with T cell-mediated therapy to overcome TCTP^high^ immune-refractory tumors.

## Results

### TCTP is associated with poor response to anti-PD-L1 therapy

**Fig. 1:**
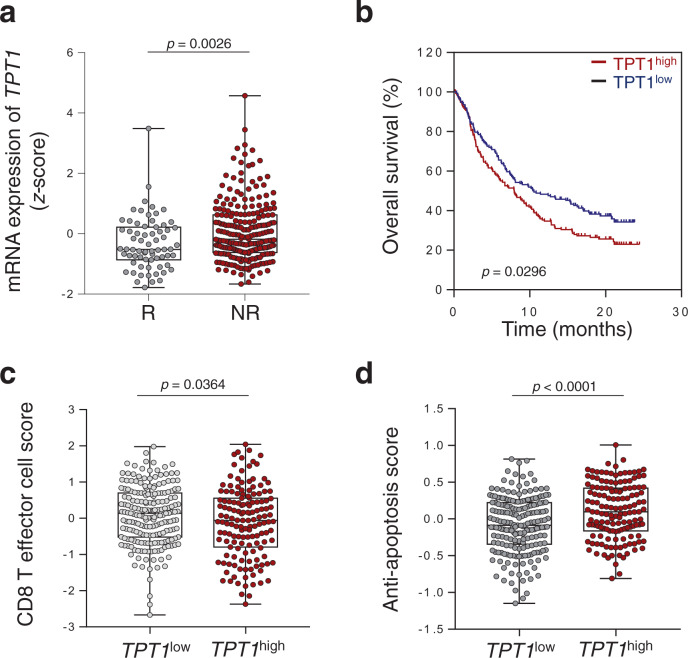
Increased expression of *TPT1* is associated with immune-refractory non-responder phenotype to anti-PD-L1 therapy. **a** Comparison of *TPT1* expression in responders (R, *n* = 68) and non-responders (NR, *n* = 230). **b** Kaplan–Meier analysis of overall survival (calculated as months to death or months to last follow-up) and median expression cutoff values for the expression level of *TPT1* (*TPT1*^high^ > average; *TPT1*^low^ < average). The *p* value was determined by Gehan–Breslow–Wilcoxon test. **c** CD8^+^ T cell signature score in *TPT1*^low^ (*n* = 201) and *TPT1*^high^ (*n* = 148) patients. **d** Anti-apoptosis signature scores in *TPT1*^low^ and *TPT1*^high^ patients. In the dot plots, the center lines indicate the medians; and the ends of the whiskers indicate the maximum and minimum values, respectively. The *p* values by two-tailed *t* test **a**, **c** and **d** are indicated. The top and bottom edges of boxes indicate the first and third quartiles, respectively. Data represent the mean ± SD. Source data are provided as a Source Data file.

To determine the clinical relevance of TCTP in response outcomes to ICB therapy, we used the transcriptome data, extracted before anti-PD-L1 therapy, from metastatic urothelial cancer (mUC) patients classified as responders (R) or non-responders (NR)^10^. From the comparative transcriptome analysis of the differentially expressed genes (DEGs), we found that the expression level of *TPT1* (gene *TPT1* encodes a TCTP) was significantly higher in the NR compared to the R (Supplementary Fig. 1a and Fig. 1a). Moreover, patients with high *TPT1* expression in their tumors had poor prognosis compared to patients with low *TPT1* expression (Fig. 1b), indicating that the expression of *TPT1* was associated with poor response to anti-PD-L1 therapy and survival outcomes of patients.

We then questioned whether TCTP is responsible for anti-PD-L1 therapy refractory properties. It has been reported that multi-gene signatures are associated with the clinical efficacy of ICB therapy^37,38^. The response outcomes to ICB therapy are predictable by evaluating the functionality of infiltrated CD8^+^ T cells that can be measured most robustly via the expression of CD8^+^ T cell signature genes^10^ (Supplementary Fig. 1b). Interestingly, *TPT1* expression was inversely correlated with T cell infiltration in the patients (Fig. 1c). Besides T cell infiltration, one of the major obstacles to successful cancer immunotherapy is the intrinsic resistance of tumor cells to CTL-mediated apoptosis that can be characterized by anti-apoptosis signature genes^39^. The anti-apoptosis signature was higher in NR compared to R and positively correlated with *TPT1* expression (Supplementary Fig. 1c and Fig. 1d). To verify whether these results are specific to mUC, we additionally analyzed other cancer cohorts received ICB therapy by using Tumor Immune Dysfunction and Exclusion (TIDE)^37^. The *TPT1* expression was correlated with poor survival and CTL score in various melanoma patients (Supplementary Fig. 2). Furthermore, we analyzed data set of patients from The Cancer Genome Atlas (TCGA)^40^. Patients with high *TPT1* expression in their tumors had poor prognosis and compared to patients with low *TPT1* expression (Supplementary Fig. 3). In addition, T cell signature scores were negatively correlated with *TPT1* expression, and anti-apoptotic signature scores were positively correlated with *TPT1* expression (Supplementary Figs. 4 and 5). Taken together, these results indicate that *TPT1* could be a biomarker in predicting the response to anti-PD-L1 therapy and clinical outcomes.

### TCTP is required for immune-refractoriness to anti-PD-L1 therapy

**Fig. 2:**
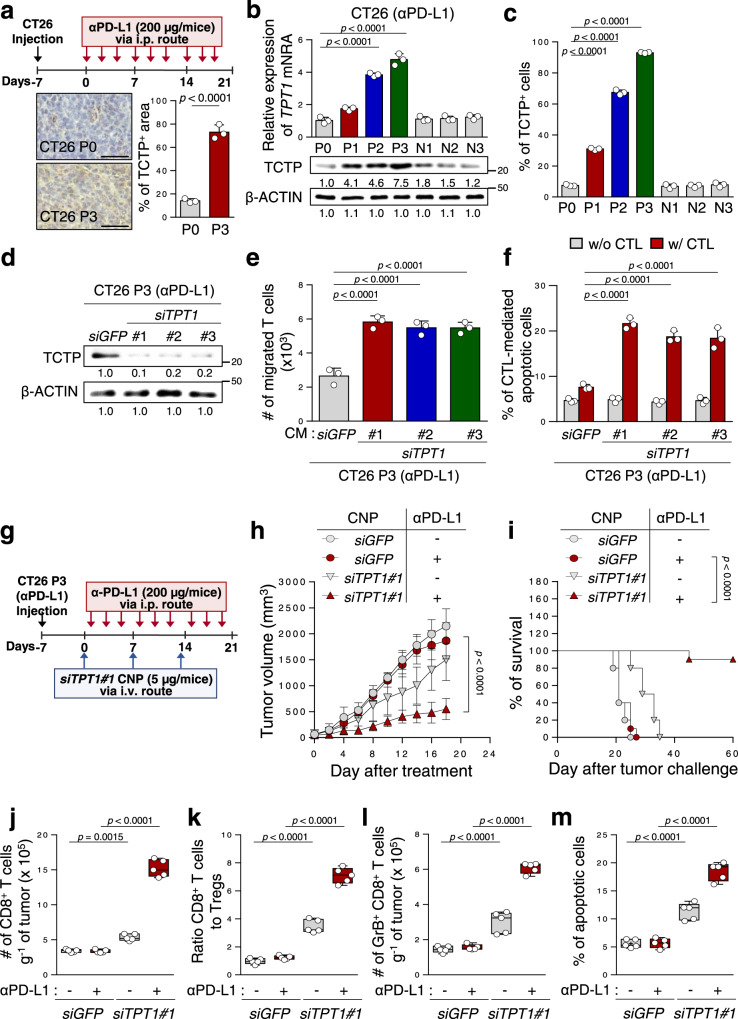
Silencing TCTP reverses tumor-intrinsic resistance to CTL-mediated killing and the non-T cell-inflamed tumor microenvironment of immune-refractory cancer. **a** Schematic of the therapy regimen in BALB/c mice implanted with CT26 P0 or P3 cells (upper). For IHC (lower), 3 mice from each group were used, and randomly selected 3 sections of each CT26 P0 or P3 tumors were analyzed. Scale bar, 50 μm. **b** *TPT1* mRNA and protein levels in CT26 cells at various stages of immune-resistance were determined by qRT-PCR and Western blot (the numbers of each blot are densitometric values). **c** The percentage of TCTP^+^ cells were analyzed by flow cytometry. **d**–**e** P3 cells were transfected with the indicated siRNAs. **d** TCTP protein were determined by Western blot analysis. **e** Transwell-based T cell chemotaxis assay by using *siGFP*- or *siTPT1#1, 2, 3*-treated CT26 P3 cell-derived conditioned media (CM). **f** CFSE-labeled tumor cells were exposed to tumor-specific CTLs and the CFSE^+^ apoptotic tumor cells was determined by flow cytometric analysis of active-caspase-3. **g** Schematic of the therapy regimen in BALB/c mice implanted with CT26 P3 cells. **h**–**m** CT26 P3 tumor-bearing mice administered *siGFP*-or *siTPT1*-CNPs with or without PD-L1 antibody. **h** Tumor growth and **i** survival of mice with the indicated reagents. **j** Flow cytometry profiles of tumor-infiltrating CD8^+^ T cells. **k** Tumor-infiltrating CD8^+^ T cell to CD4^+^, Foxp3^+^ Treg cell ratio. **l** The absolute number of granzyme B^+^ cells in CD8^+^ T cells. **m** The frequency of apoptotic cells in the tumors. For the in vivo experiments, 10 mice from each group were used, and randomly selected 5 samples were analyzed **j**–**m**. All in vitro experiments were performed in triplicate. The *p* values by two-way ANOVA **f**, **h**, one-way ANOVA **b**, **c**, **e** and **j**–**m**, and the log-rank (Mantel–Cox) test **i** are indicated. In the box plots, the top and bottom edges indicate the first and third quartiles; the center lines indicate the medians; and the whiskers ends indicate the maximum and minimum, respectively. The data represent the mean ± SD. Source data are provided as a Source data file.

To explore the mechanisms for the refractory phenotypes of tumors to ICB therapy, we developed an ICB-refractory CT26 P3 tumor model generated from an ICB-susceptible parental cell line, CT26 P0, through three rounds of in vivo selection by anti-PD-L1 therapy (Fig. 2a). While anti-PD-L1 antibody treatment successfully retarded tumor growth and prolonged mouse survival in CT26 P0 tumor-bearing mice, there were no remarkable therapeutic effects in CT26 P3 tumor-bearing mice (Supplementary Fig. 6a, b). Consistently, relative to CT26 P0 tumors, P3 tumors exhibited non-T cell-inflamed immune phenotypes, as evidenced by decreased levels of overall CD8^+^ T cells, the ratio of CD8^+^ T cells to T regs and tumor-reactive CD8^+^ T cells producing granzyme B (GrB), (Supplementary Fig. 6c–e), and anti-apoptotic phenotypes (Supplementary Fig. 6f). These refractory phenotypes of CT26 P3 tumors were not reversed by PD-L1 blockade, although anti-PD-L1 therapy significantly induced T cell-inflamed immune phenotypes and apoptotic cell death in the CT26 P0 tumors (Supplementary Fig. 6f). These data indicate that the refractoriness to anti-PD-L1 therapy shown in patients were conserved in our ICB-refractory tumor model.

Next, we attempted to further characterize the ICB-refractory tumor model. Having explored that tumor cells could regulate T cell trafficking^10,17^, we performed an in vitro Transwell-based chemotaxis assay and found that CT26 P3 cells had a much lower capacity to recruit the T cells compared to CT26 P0 cells (Supplementary Fig. 7a). Furthermore, as T cells are trafficked to tumors by following a chemokine gradient^41,42^, we tested T cell chemotaxis by using conditioned media (CM) derived from CT26 P0 or P3 cells and observed that CT26 P3-derived CM markedly reduced T cell chemotaxis compared to CM from P0 cells (Supplementary Fig. 7b). These results suggest that ICB-refractory CT26 P3 cells could inhibit T cell infiltration by decreasing the soluble factors responsible for T cell chemotaxis. Consistent with the anti-apoptotic property of the ICB-refractory tumor cells in vivo, CT26 P3 cells were refractory to apoptotic death by cognate CTLs specific to AH-1, an immunodominant H-2Ld-restricted peptide (gp70_413–423_) from CT26 in vitro, whereas the CT26 P0 cells remained sensitive to cognate CTLs (Supplementary Fig. 7c). It had been well documented that tumor cells escape CTL attack through tumor-intrinsic events, such as the loss of antigen or MHC class I, and resistance to apoptosis^3^. We found no difference in the antigen or MHC class I expression between the P0 and P3 cells, and the percentage of CTL effector cytokine (IFN-γ) production T cells incubated with P0 or P3 cells (Supplementary Fig. 7d–f). Importantly, the CT26 P3 cells were resistant to in vitro liposomal delivery of GrB, which is a key component of CTL-mediated apoptosis (Supplementary Fig. 7g), indicating that the P3 cells were resistant to apoptotic death by CTLs, regardless of the T cell recognition and functional capacity of the CTLs. Our findings suggest that intrinsic tumor properties could determine ICB-refractory phenotypes.

To characterize the role of TCTP in ICB-refractory properties, we measured the levels of TCTP mRNA and protein in different rounds of selection by anti-PD-L1 therapy (P0 to P3) or control IgG treatment (N1 to N3). The percentage of TCTP^+^ area in P0 tumor was about 13%, those of P3 tumors was more than 75% (Fig. 2a). Furthermore, mRNA, protein levels of TCTP, and the percentage of TCTP^+^ cells were stepwisely increased from P0 to P3 (Fig. 2b, c). To directly link TCTP to the ICB-refractory phenotypes of CT26 P3 tumor cells, we silenced *TPT1* in CT26 P3 cells using siRNAs (Fig. 2d). Notably, T cell migration was increased when incubated with CM derived from *siTPT1*-transfected P3 cells, compared to *siGFP*-transfected P3 cells (Fig. 2e). While there was no alteration in the percentage of apoptotic tumor cells in *siTPT*-treated and *siGFP*-treated P3 tumor cells without CTLs, *TPT1* knockdown sensitizes tumor cells to CTL-mediated apoptosis (Fig. 2f). To further verify whether knockdown of TCTP in tumor cells increase the spontaneous apoptosis of tumor cells as much as CTLs-mediated apoptosis, we compared apoptosis of tumor cells with or without CTLs from 24 to 96 h after TCTP knockdown. The increase in apoptosis of *siTPT1*-treated tumor cells with CTLs was much greater than that in *siTPT1*-treated tumor cells without CTLs (Supplementary Fig. 8). Therefore, we conclude that knockdown of TCTP sensitizes immune-refractory tumor cells to CTL-mediated cell death.

On the basis of our in vitro observations, we reasoned in vivo silencing of *TPT1* could reverse the refractory phenotypes of CT26 P3 tumors to anti-PD-L1 therapy. We treated CT26 P3-bearing mice with anti-PD-L1 therapy along with intravenously administered chitosan nanoparticles (CNPs) carrying *siTPT1 or siGFP* for the in vivo delivery of siRNAs to tumors^12^ (Fig. 2g). While anti-PD-L1 therapy alone had no effect on tumor growth, the anti-PD-L1 antibody with *siTPT1*-CNPs profoundly retarded tumor growth (Fig. 2h), and prolonged the survival (Fig. 2i). Moreover, to confirm whether the CD8^+^ CTLs are a major intervention in therapeutic effect of combination therapy group, we depleted CD8^+^ T cells by using anti-CD8^+^ antibody. Therapeutic effects of combining *siTPT1*-CNPs and anti-PD-L1 almost reversed upon CD8^+^ T cell depletion (Supplementary Fig. 9). Therefore, we conclude that enhanced immunotherapeutic effects of combining *siTPT1*-CNPs and anti-PD-L1 antibody were mainly dependent to CD8^+^ T cells. Furthermore, the number of functional CD8^+^ T cells infiltrating the tumor and apoptotic tumor cells was significantly increased in the combined treatment compared to either treatment alone (Fig. 2j–m). Altogether, our data indicate that targeting TCTP could improve the therapeutic efficacy of anti-PD-L1 via reversing immune-refractory phenotypes.

### Ectopic expression of TCTP promotes immune-refractory phenotypes

**Fig. 3:**
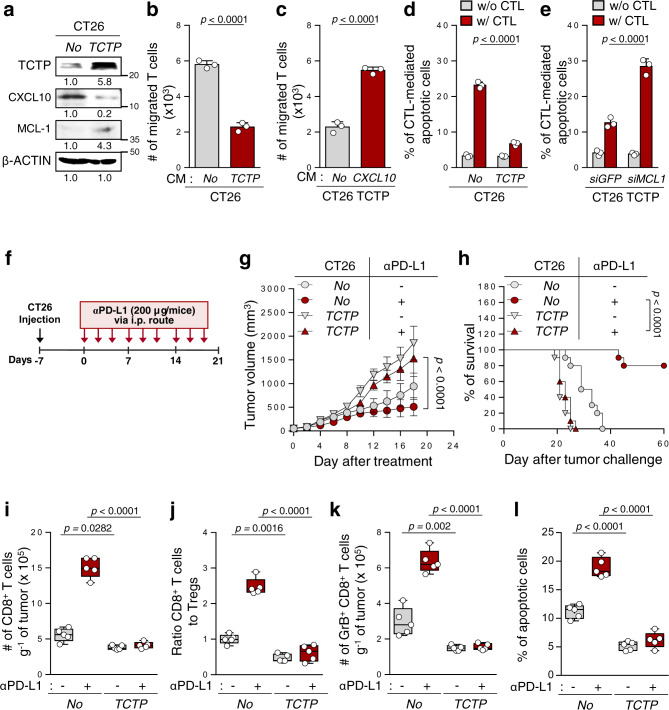
Overexpression of TCTP changes the properties of tumor cells to those of an immune-refractory tumor. **a**, **b** and **d** CT26 cells were stably transfected with empty vector (No) or TCTP. **a** The levels of TCTP, CXCL10, and MCL-1 were analyzed by Western blots. **b** T cell chemotaxis assays were performed using CT26 No or CT26 TCTP cell-derived CM. **c** The migrated T cells were counted after incubation with empty vector- or *CXCL10*-transfected CT26 TCTP cell-derived CM. **d** and **e** CFSE-labeled tumor cells were incubated with tumor-specific CTLs and the frequency of CFSE^+^ apoptotic tumor cells was determined by flow cytometric analysis of active-caspase-3. **f** Schematic of the therapy regimen in BALB/c mice implanted with CT26 No or CT26 TCTP cells. **g**–**l** Tumor-bearing mice administered treatment or not treated with the PD-L1 antibody. **g** Tumor growth and **h** survival of mice inoculated with CT26 No or TCTP cells treated with or without PD-L1 antibody. **i** Flow cytometry profiles of tumor-infiltrating CD8^+^ T cells. **j** Tumor-infiltrating CD8^+^ T cell to CD4^+^, Foxp3^+^ Treg cells ratio. **k** The absolute number of granzyme B^+^ to tumor-infiltrating CD8^+^ T cells. **l** The frequency of apoptotic cells in the tumors treated with the indicated reagents. All in vitro experiments were performed in triplicate For the in vivo experiments, 10 mice from each group were used, and randomly selected 5 samples were analyzed **i**–**l**. The numbers below the blot images indicate the expression as measured fold change **a**. The *p* values by by two-tailed *t* test **b**, **c**, one-way ANOVA **i**–**l**, two-way ANOVA **d**, **e**, **g**, and the log-rank (Mantel–Cox) test **h** are indicated. In the box plots, the top and bottom edges of boxes indicate the first and third quartiles, respectively; the center lines indicate the medians; and the ends of whiskers indicate the maximum and minimum values, respectively. The data represent the mean ± SD. Source data are provided as a Source data file.

Given the crucial role of the TCTP in ICB-refractory tumors, we examined whether TCTP expression alone could promote the immune-refractory phenotypes. The overexpression of *TPT1* in CT26 P0 cells reduced T cell chemotaxis and increased resistance to CTL-mediated apoptosis without T cell recognition problems and functional capacity change of CTLs (Supplementary Fig. 10 and Fig. 3a, b, d). In an effort to elucidate the a key molecule in the TCTP-mediated inhibition of T cell chemotaxis, we noted that chemokines play integral roles in T cell trafficking^42^. Among the chemokines, the level of *CXCL10* was significantly decreased in CT26 P3 cells, compared to CT26 P0 cells (Supplementary Fig. 11a). In this regard, the increased capacity to recruit the T cells after *TPT1* knockdown in CT26 P3 cells was reversed by the neutralization of secreted CXCL10 with monoclonal antibodies (Supplementary Fig. 11b). Notably, the level of CXCL10 was significantly decreased upon *TPT1* overexpression (Fig. 3a), and restoring *CXCL10* expression in TCTP-ectopically-expression CT26 P0 cells, CT26 TCTP cells, reversed T cell chemotaxis (Fig. 3b, c), indicating an important role of CXCL10 in the property mediated by TCTP. For the TCTP-mediated anti-apoptotic response to CTLs, we noted an increase in anti-apoptotic protein MCL-1, a signature molecule rendering tumor cells resistant to CTL-induced killing in previous studies^12,14,19^, in CT26 P3 cells as well as CT26 TCTP cells, relative to P0 or CT26-no cells, respectively (Supplementary Fig. 11c and Fig. 3a). The knockdown of MCL-1 restored the susceptibility of CT26 P3 or CT26 TCTP cells to CTL-mediated apoptosis (Fig. 3d, e and Supplementary Fig. 11d). Thus, we concluded that CXCL10 and MCL-1 were key mediators of the TCTP-induced immune-refractory phenotypes. Consistent with the in vitro results, TCTP overexpression conferred a poor response to anti-PD-L1 therapy in vivo (Fig. 3f–h). This was accompanied by decreased numbers of tumor-infiltrated CD8^+^ T cells, and the ratio of CD8^+^ T cells to T regs and tumor-reactive CD8^+^ T cells (Fig. 3i–k), as well as the apoptotic cell death of tumor cell populations (Fig. 3l). Given these results, we concluded that TCTP itself was sufficient to promote the non-T cell-inflamed immune-phenotype and resistance of tumor cells to CTL killing, thereby contributing to anti-PD-L1 therapy resistance.

### TCTP^+^ cells which are enriched by CTL-mediated immune selection play an important role in immune-refractory phenotype of tumor cells

**Fig. 4:**
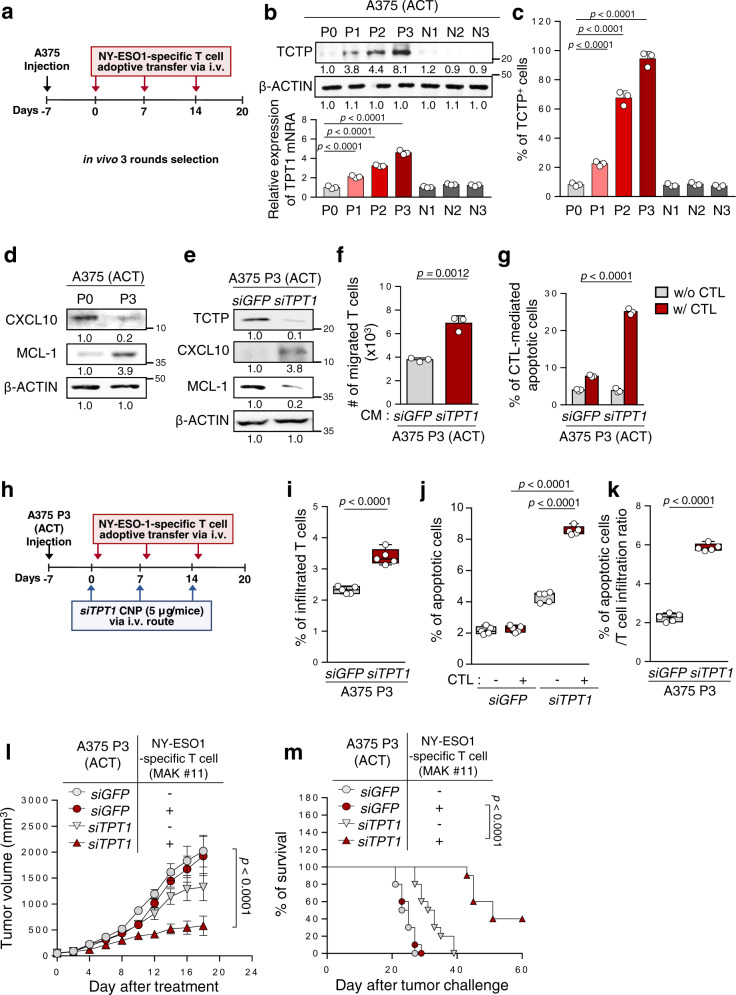
CTL-mediated immune selection enriches TCTP^+^ immune-refractory tumor cells. **a** Schematic of the therapy regimen in NOD/SCID mice implanted with A375 P0 or P3 cells. **b** *TPT1* mRNA and protein levels at various stages of immune-resistance A375 cells were determined by qRT-PCR and Western blot. **c** TCTP^+^ tumor cells were analyzed by flow cytometry. **d** The protein levels of CXCL10 and MCL-1 were analyzed. **e**–**g** P3 cells were transfected with the indicated siRNAs. **e** The protein levels of TCTP, CXCL10, and MCL-1 were determined by Western blot. **f** Transwell-based T cell chemotaxis assays were performed using *siGFP*- or *siTPT1*-treated A375 P3 cell-derived CM. **g** CFSE^+^ apoptotic tumor cells (active-caspase-3^+^) was determined by flow cytometry. **h** Schematic of the therapy regimen in NOD/SCID mice implanted with A375 P3 cells. **i**–**m** P3 tumor-bearing mice administered *siGFP*-or *siTPT1*-CNPs with or without NY-ESO1-specific T cell adoptive transfer. **i** Flow cytometry profiles of CFSE^+^ adoptively transferred NY-ESO1-specific T cells. **j** Active-caspase-3^+^ apoptotic cells in the tumors. **k** The frequency of apoptotic cells in the tumor relative to the NY-ESO1-specific T cells migrated to the tumor. **l** Tumor growth and **m** survival of mice inoculated with A375 P3 cells treated with the indicated reagents. All in vitro experiments were performed in triplicate. For the in vivo experiments, 10 mice from each group were used, and randomly selected 5 samples were analyzed **i**–**k**. The numbers below the blot images indicate fold change **b**, **d**, and **e**. The *p* values by one-way ANOVA **b**, **c**, **j**, and two-tailed *t* test **f**, **i**, **k**, and two-way ANOVA **g**, **l**, and the log-rank (Mantel–Cox) test **m** are indicated. In the box plots, the top and bottom edges of boxes indicate the first and third quartiles; the center lines indicate the medians; and the ends of whiskers indicate the maximum and minimum, respectively. The data represent the mean ± SD. Source data are provided as a Source data file.

Previously, we found that CTL-mediated immune selection promoted the enrichment of a subset of cells with immune-refractory properties^12,14^. As tumor antigen-specific CTLs are key effectors in anti-PD-L1 therapy, we reasoned that increased TCTP expression under anti-PD-L1 therapy is due to immune selection imposed by CTLs. To test this possibility, we chose the A375 human melanoma cells, the most typical cancer for the clinical application of adoptive CD8^+^ T cell transfer therapy (ACT), and established an ACT-refractory A375 P3 model from parental A375 P0 cells by selection with NY-ESO1-specific CD8^+^ T cells (MAK #11) in vivo (Fig. 4a). While the adoptive transfer of NY-ESO1-specific CTLs significantly retarded tumor growth and prolonged mouse survival in A375 P0 tumor-bearing NOD/SCID mice, there was no remarkable therapeutic effects in the A375 P3 tumor-bearing mice (Supplementary Fig. 12). Interestingly, relevant to the ICB-refractory tumor model, A375 P3 cells had immune-refractory properties, including a lower capacity to induce T cell migration and resistance to CTL-mediated killing (Supplementary Fig. 13). Indeed, the levels of TCTP mRNA and protein were increased in different rounds of CTL-mediated immune selection (Fig. 4b) which was likely due to the enrichment of TCTP^+^ cells during the ACT of the MAK #11 clone, as evidenced by an increased proportion of TCTP^+^ cells from around 8.9% in the A375 P0 cells to around 94.9% in the A375 P3 cells (Fig. 4c). Changes in MCL-1 and CXCL10 protein levels were also observed in the A375 P3 cells compared to the P0 cells (Fig. 4d).

Notably, TCTP knockdown in A375 P3 cells increased T cell migration and sensitized tumor cells to CTL-mediated killing, which was accompanied by profound changes in CXCL10 and MCL-1 (Fig. 4e–g). To demonstrate the therapeutic value of inhibiting TCTP, we inoculated A375 P3 cells into NOD/SCID mice and intravenously administered *siTPT1*- or *siGFP*-CNPs (Fig. 4h). The infiltrated functional T cells and apoptotic tumor cells were increased in the *siTPT1*-treated A375 P3 tumors compared to the *siGFP*-treated A375 P3 tumors (Fig. 4i–j). As shown in Fig. 4k, relative to adoptive T cell transfer efficacy, the percentage of apoptotic cells was increased in the tumors of *siTPT1*-treated mice compared to *siGFP*-treated mice, indicating that the combined therapeutic effects of targeting TCTP and ACT were affected by both induced CTL trafficking to the tumor and increased CTL-mediated apoptotic tumor cells. Consistently, combined therapy with *siTPT1-*CNPs and ACT profoundly retarded tumor growth (Fig. 4l) and prolonged the survival of the mice (Fig. 4m). Together, our data indicate that the enrichment of TCTP^+^ immune-refractory tumor cells under CTL-mediated immune selection could cause the tumor phenotypes refractory to ACT therapy. Therefore, therapeutic strategies targeting TCTP could reverse immune-refractory phenotypes, thereby improving the efficacy of ACT and ICB therapy.

### TCTP activates EGFR-AKT signaling by phospho-dependent binding with Na, K ATPase

**Fig. 5:**
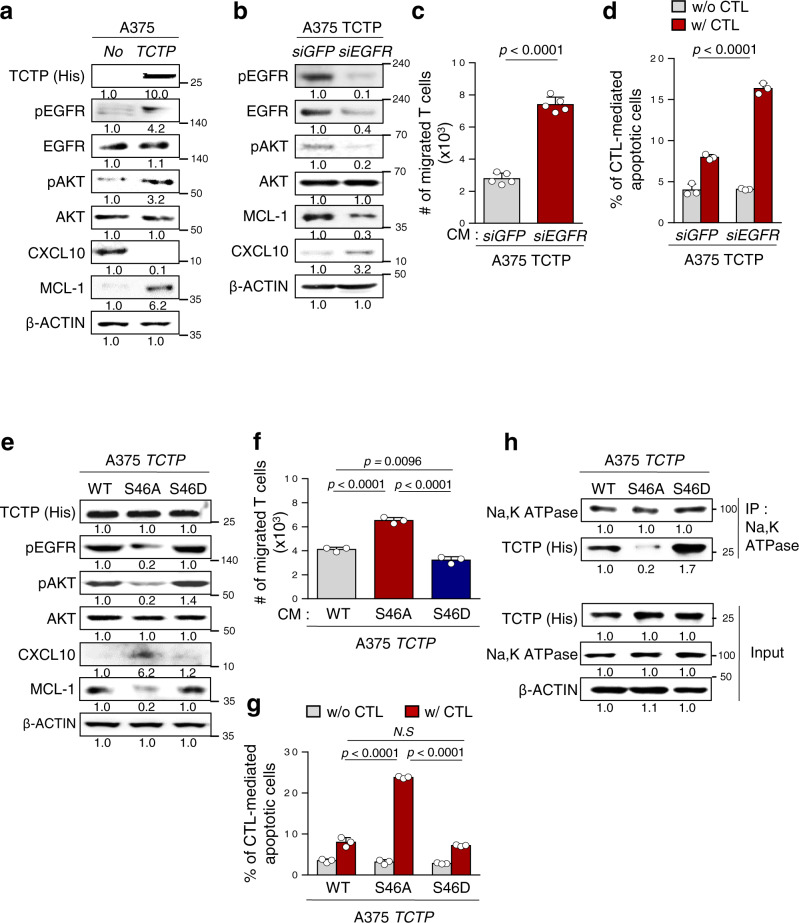
TCTP phosphorylation is crucial to activating the EGFR/AKT signaling pathway via binding with Na, K ATPase. **a** The protein levels of TCTP, EGFR, pEGFR, pAKT, AKT, MCL-1, and CXCL10 were measured by Western blot analysis. **b** The protein levels of pEGFR, EGFR, pAKT, AKT, MCL-1, and CXCL10 in *SiGFP*- or *siEGFR-*treated A375 TCTP cells were analyzed by Western blots. **c** *SiGFP*- or *siEGFR-*treated A375 TCTP cells CM was added to the lower chamber, and CD8+ T cells were plated in the upper chamber. The T cells migrated into the lower chamber media were collected after 6 h and counted. **d** CFSE-labeled tumor cells were exposed to NY-ESO1-specific CTLs and the frequency of CFSE + apoptotic tumor cells was determined by flow cytometric analysis of active-caspase-3. **e**–**h** A375 cells were transfected with FLAG-TCTP wild type (TCTP), FLAG-TCTP S46A mutant, or FLAG-TCTP S46D mutant. **e** Activation of EGFR, AKT signaling and expression of MCL-1 and CXCL10 were analyzed by western blot assays. **f** T cell chemotaxis assays were performed by using the indicated tumor cell-derived CM. **g** Active-caspase-3^+^ apoptotic tumor cells were analyzed by flow cytometry after incubation with CTLs. **h** Cell lysates were immunoprecipitated with anti-Na, K ATPase antibody. The immunoprecipitated proteins were analyzed by Western blot assays. The data are representative of three separate experiments. The numbers below the blot images indicate the expression as measured fold change **a**, **b**, **e**, and **h**. The *p* values by two-tailed *t* test **c**, two-way ANOVA **d**, **g**, and one-way ANOVA **f** are indicated. *N.S*, not significant. The error bars represent mean ± SD. Source data are provided as a Source data file.

We next attempted to elucidate the signaling pathway by which TCTP conferred the immune-refractory phenotypes. To gain insight into the TCTP-dependent signaling changes and biological process, we analyzed functional enrichment analysis of DEGs using Enrich R system in upregulated or downregulated gene set from *TPT1*^high^ patients transcriptome data compared with those of *TPT1*^low^ patients. In Gene Ontology (GO) analysis, upregulated genes were positively correlated with anti-apoptotic process, and downregulated genes were positively related with T cell chemotaxis (Supplementary Fig. 14a). In addition, genes upregulated in *TPT1*^high^ patients were mostly related to PI3K-AKT signaling pathways (Supplementary Fig. 14b). We previously found that hyperactivation of the EGFR-AKT pathway was closely linked to the immune escape of tumor cells^43^. In addition, another group revealed that TCTP activates the EGFR signaling pathway via binding to the Na, K ATPase α1 subunit^29^. Notably, TCTP overexpression increased the phosphorylation of both EGFR and AKT, and reduced T cell chemotaxis and CTL susceptibility, which were accompanied by CXCL10 downregulation and MCL-1 upregulation (Supplementary Fig. 15 and Fig. 5a). Conversely, the knockdown of EGFR in A375 TCTP cells robustly dampened the levels of phosphorylated AKT and MCL-1, but increased CXCL10 levels (Fig. 5b), demonstrating activation of the EGFR-AKT-MCL-1/CXCL10 axis by TCTP. Consistently, loss of EGFR markedly increased T cell chemotaxis and susceptibility to CTLs in A375 TCTP cells (Fig. 5c, d). Taken together, we concluded that the hyperactivation of EGFR signaling by TCTP drove the immune-refractory phenotypes.

Although a previous study revealed that the binding of TCTP to the Na, K ATPase α1 subunit was important for EGFR activation^29^, it is unclear how TCTP binds to the Na, K ATPase α1 subunit. It has been reported that TCTP phosphorylation at serine 46 by polo-like kinase 1 (PLK1) is important in the interactome and biological function of TCTP^44,45^. Indeed, we found that the phosphorylation level at TCTP Ser46 was increased in ACT-refractory P3 cells compared to P0 cells (Supplementary Fig. 16a). It seems that the increase in phosphorylated TCTP (pTCTP) was due to the upregulation of total TCTP rather than changes in the quantity of PLK1 protein, as evidenced by identical pTCTP to TCTP ratios in the A375 P0 and P3 cells. To gain insight into the phosphorylation status in TCTP-induced hyperactivation of EGFR-AKT signaling, we treated A375 P3 cells with PLK1 inhibitor (BI2536) to decrease the level of pTCTP. Intriguingly, treatment with BI2536 attenuated the pTCTP and EGFR-AKT axis, and effectively reversed the immune-refractory properties of A375 P3 cells (Supplementary Fig. 16b–d), suggesting that the phosphorylation of TCTP by PLK1 was crucial for EGFR-AKT signaling as well as the immune-refractory properties. We further confirmed it by using two mutant forms of TCTP, including a phospho-loss mutant TCTP (TCTP S46A) and a phospho-mimic mutant TCTP (TCTP S46D). Similar to TCTP WT, TCTP S46D transfection into A375 P0 cells led to the activation of the EGFR-AKT signaling pathway, and promoted the immune-refractory properties of the tumor cells (Fig. 5e–g). In contrast, TCTP S46A failed to reflect the biochemical and functional properties of TCTP WT, demonstrating the important role of phosphorylation in these properties mediated by TCTP. The binding of TCTP to the Na, K ATPase α1 subunit contributes to the activation of the EGFR signaling pathway^29^. Interestingly, TCTP WT or S46D co-precipitated with Na, K ATPase α1, whereas TCTP S46A did not (Fig. 5h), indicating a phosphorylation-dependent interaction between TCTP and Na, K ATPase α1. Therefore, our data reveal that the phospho-dependent binding of TCTP to Na, K ATPase leads to the activation of EGFR-AKT signaling, indicating that blocking TCTP phosphorylation could be an additional combination strategy with T cell-mediated therapy.

### Inhibition of TCTP with DHA increases T cell-mediated tumor cell killing, and T cell chemotaxis capacity of TCTP^high^ tumor cells

**Fig. 6:**
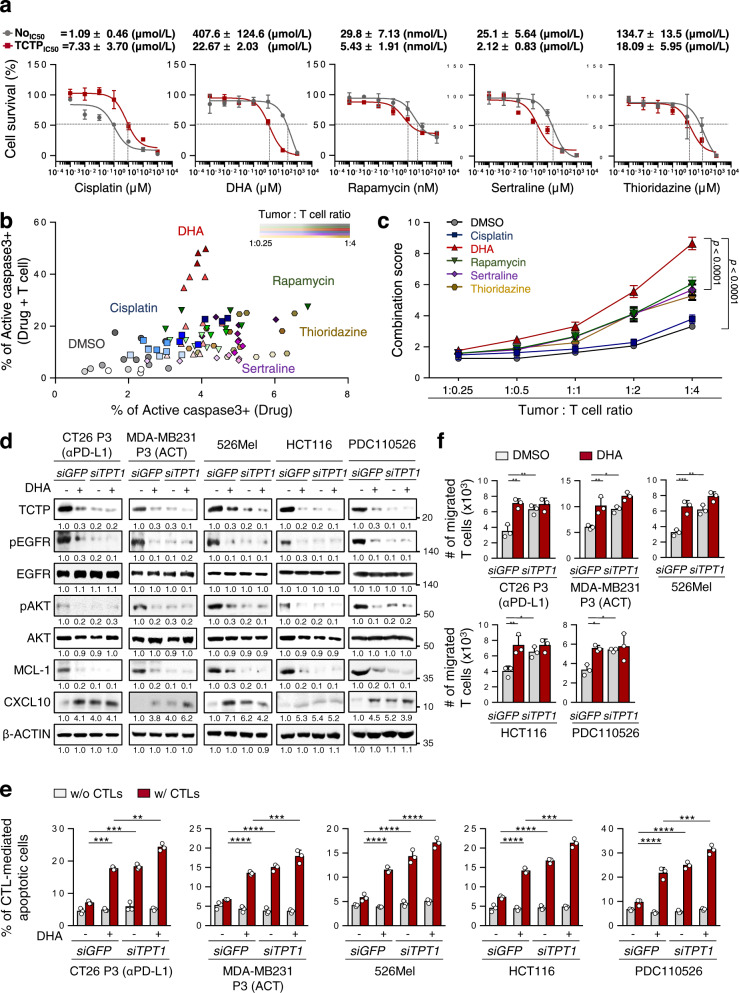
Inhibition of TCTP by DHA sensitizes TCTP^high^ tumor cells to T cell-mediated killing and increases T cell migration. **a** CT26 No and TCTP cells were treated with the indicated concentrations of cisplatin, DHA, rapamycin, sertraline, and thioridazine for 24 h. Cell viability was measured by the trypan blue exclusion assay, and then the concentrations causing a 50% decrease in cell viability (IC50 values) were determined. **b** CT26 TCTP cells were treated with the indicated agents, and incubated with tumor-specific CTLs at the indicated tumor: T cell ratio. The percentage of active-caspase-3^+^ apoptotic tumor cells was analyzed by flow cytometry. **c** The combination score was calculated based on changes in the percentage of apoptosis in drug-treated tumor cells with or without CTLs. Combination score = (% of active-caspase 3^+^ tumor cells by drug and CTLs) / (% of active-caspase 3+ tumor cells by drug). **d** *SiGFP*- or *siTPT1*-treated CT26 P3, MDA-MB231, 526Mel, HCT116, and PDC110526 cells were treated with DMSO or DHA. The levels of TCTP, pEGFR, EGFR, pAKT, AKT, MCL-1, CXCL10, and β‐ACTIN were analyzed by western blots. **e** The percentage of CTL-mediated anti-apoptotic tumor cells was determined by flow cytometry. **f** T cell chemotaxis assays were performed using DMSO or DHA-treated *siGFP*- or *siTPT1*-treated tumor cell CM. The data are representative of three separate experiments. The numbers below the blot images indicate the expression as measured as fold change **d** The error bars represent mean ± SD. *p < 0.05,**p < 0.01, ***p < 0.001, and ****p < 0.0001. The *p* values by two-way ANOVA **c**, **e**, **f** are indicated. Source data and exact *p* values are provided as a Source data file.

Having explored that targeting TCTP could be a potential therapeutic strategy to overcome immunotherapy refractoriness, we aimed to screen clinically-actionable drugs that could target TCTP to reverse the immune-refractory phenotypes of TCTP^high^ tumor cells. It has been suggested that a number of drugs such as DHA, rapamycin, sertraline, and thioridazine had an inhibitory effect on TCTP function^33–36^. Indeed, while CT26 TCTP cells were refractory to cisplatin as reported previously^28^, these cells were more susceptible to TCTP-targeting agents, especially to DHA, a clinically available drug to treat malaria (CT26 No IC50 = 407.6 uM, CT26 TCTP IC50 = 22.67 μM, about 20-fold) (Fig. 6a). In line with this observation, TCTP^high^ ICB-refractory CT26 P3 cells were also more sensitive to DHA than ICB-susceptible CT26 P0 cells (Supplementary Fig. 17). To further investigate the effect of each drug on sensitizing TCTP^high^ tumor cells to CTL-mediated killing, CT26 TCTP tumor cells were incubated with CTLs at various tumor cell–T cell ratios after treatment with a sublethal dose of each drug. Compared to DMSO or cisplatin, TCTP-targeting drugs augmented CTL-mediated cytotoxicity in a synergistic fashion (Fig. 6b and Supplementary Fig. 18). To quantify the synergistic effects of treatment of each drugs with CTLs, a combination score was calculated based on changes in the percentage of apoptosis in drug-treated tumor cells with or without CTLs. From this analysis, we found that the score of the combination with DHA was remarkedly higher than other drugs at all ratios (Fig. 6c). Given these data, we concluded that DHA was the most effective drug to reverse the immune-refractory phenotypes of TCTP^high^ tumor cells.

To verify the phenotypic effects of DHA in multiple types of TCTP^high^ tumor cells, we further employed previously established ACT-refractory MDA-MB-231 P3 cells^19^ and human cancer cells 526Mel, HCT116, and pancreas primary tumor cell PDC110526^46^ which expressed TCTP at high level (Supplementary Fig. 19). Consistently, the knockdown of TCTP robustly dampened the EGFR-AKT-MCL-1/CXCL10 pathway across all tested cells (Fig. 6d). Notably, DHA treatment decreased TCTP levels, and resulted in the similar effects on the level of these molecules compared to treatment with *siTPT1* (Fig. 6d). Importantly, both *siTPT1-* and DHA-treated tumor cells were more susceptible to CTL-mediated apoptosis, and they also had increased T cell chemotaxis capacity compared to *siGFP-* or DMSO-treated control cells, respectively (Fig. 6e, f). These results demonstrated that the biochemical and functional properties of the TCTP axis were conserved across multiple types of cancer cells and that impeding TCTP signaling with DHA is a widely applicable strategy for controlling immune-refractory TCTP^high^ cancer cells.

### Targeting TCTP by using DHA reverses resistance to ACT therapy and anti-PD-L1 therapy

**Fig. 7:**
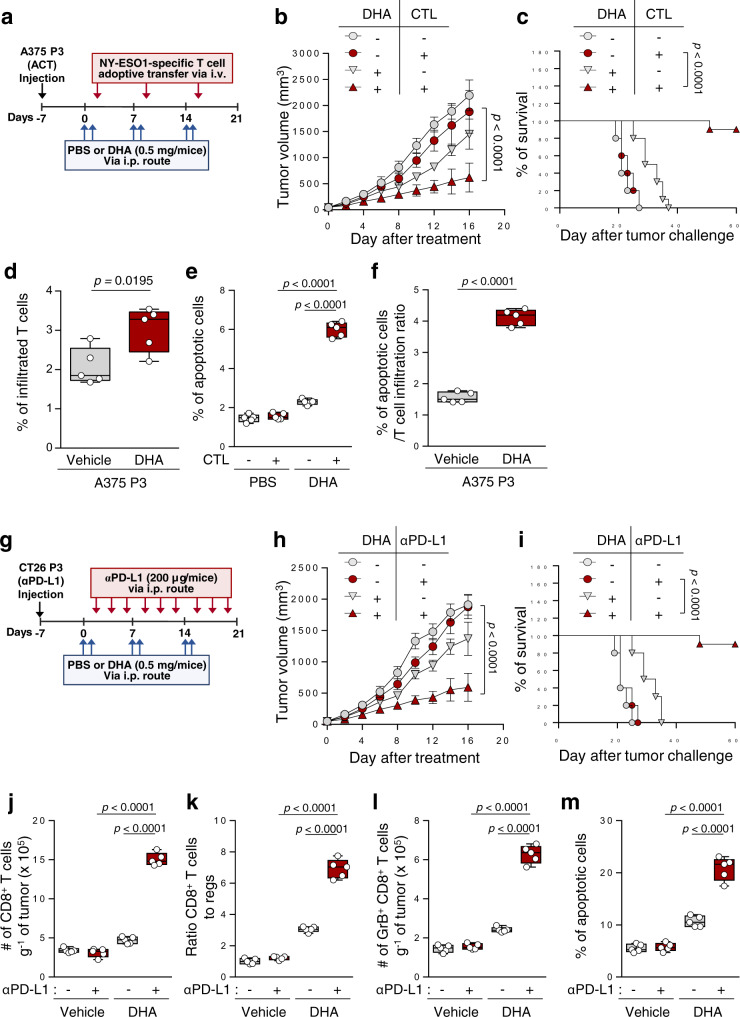
TCTP inhibition by DHA renders the tumor susceptible to T cell-mediated therapy. **a**–**f** A375 P3 tumor-bearing NOD/SCID mice administered DHA with or without NY-ESO1-specific T cell adoptive transfer treatment. **b** Tumor growth and **c** survival of mice inoculated with A375 P3 cells treated with the indicated reagents. **d** Flow cytometry profiles of CFSE^+^ adoptively transferred NY-ESO1-specific T cells. **e** The percentage of active-caspase-3^+^ apoptotic cells in the tumors treated with the indicated reagents. **f** The percentage of apoptotic cells relative to the efficacy of T cell trafficking. **g** Schematic of the therapy regimen in BALB/c mice implanted with CT26 P3 cells. **h**–**m** CT26 P3 tumor-bearing mice administered DHA with or without PD-L1 antibody treatment. **h** Tumor growth and **i** survival of mice inoculated with CT26 P3 cells treated with the indicated reagents. **j** Flow cytometry profiles of tumor-infiltrating CD8^+^ T cells. **k** Tumor-infiltrating CD8^+^ T cell to CD4^+^, Foxp3^+^ Treg cell ratio. **l** The absolute number of granzyme B^+^ cells to tumor-infiltrating CD8^+^ T cells. **m** The frequency of apoptotic cells in the tumors treated with the indicated reagents. For the in vivo experiments, 10 mice from each group were used, and randomly selected 5 samples were analyzed **d**–**f**, and **j**–**m**. The *p* values by two-way ANOVA **b** and **h**, and log-rank (Mantel–Cox) test **c** and **I**, two-tailed *t* test **d**, **f**, and one-way ANOVA **e**, **j**–**m** are indicated. In the box plots, the top and bottom edges of boxes indicate the first and third quartiles, respectively; the center lines indicate the medians; and the ends of whiskers indicate the maximum and minimum values, respectively. Data represent the mean ± SD. Source data are provided as a Source data file.

Given our observations in vitro, we reasoned that the in vivo administration of DHA should reverse resistance of TCTP^high^ tumor cells to T cell-mediated therapy. To test this possibility, ACT-refractory A375 P3 tumor-bearing NOD/SCID mice were treated cognate NY-ESO1-specific CTLs with or without DHA (Fig. 7a). While CTLs alone had no effect on tumor growth, dual therapy with CTLs and DHA retarded tumor growth (Fig. 7b), and prolonged survival of the mice compared to the other groups (Fig. 7c). The proportion of NY-ESO1-specific CTLs in the tumors was increased in the tumors of DHA-treated mice compared to those in control group mice (Fig. 7d), and the overall cytotoxic effects of these CTLs were greater after treatment with DHA relative to the control, as indicated by the percentage of apoptotic cells in the tumor populations (Fig. 7e, f).

Next, we expanded the preclinical therapeutic value of DHA in ICB therapy. To do this, ICB-refractory CT26 P3 tumor-bearing mice were administered anti-PD-L1 antibody alone or combined with DHA (Fig. 7g). Compared to treatment with anti-PD-L1 or DHA alone, combined therapy with anti-PD-L1 and DHA showed a remarkable therapeutic effect in CT26 P3 tumor-bearing mice (Fig. 7h). Importantly, while 90% of the mice that received both the anti-PD-L1 blockade agent and DHA survived, all of the mice in the other groups died (Fig. 7i). In addition, the numbers of infiltrated functional CD8^+^ T cells and the cytotoxic effect of these CTLs were significantly higher in the co-treated mice group than in the other mice groups (Fig. 7j–m). It is notable that TCTP expression is commonly increased in various type of immune-refractory tumor model to T cell-mediated therapy (Figs. 2b, 4b and Supplementary Fig. 19), and targeting TCTP by using DHA in TCTP^high^ tumor cells including immune-refractory tumor cells decreased immune-refractory TCTP-EGFR-MCL-1/CXCL10 signaling pathway and phenotypes (Fig. 6d–f). Taken together, these results indicate that targeting TCTP by using the actionable drug, DHA, is potential combinational strategy enhancing the response to various type of T cell-mediated therapy such as ACT and ICB therapy to treat multiple type of TCTP^high^ cancer.

## Discussion

The main purpose of cancer immunotherapy is to initiate or reinitiate a self-sustaining cycle of anti-cancer immunity with a durable clinical response. However, the majority of patients fail to amplify and propagate the cycle, demonstrating therapeutic resistance. Thus, elucidating a resistant factor that tackles one or more steps of the cancer immunity cycle is needed to overcome immunotherapy resistance. In this study, we proposed that TCTP acted as an immune-resistant factor in tumors by disrupting multiple steps of the cycle, such as the CTL-mediated killing of tumor cells and T cell infiltration into tumors. Tumor cell killing by CTLs is known as a crucial final step to tumor regression. Moreover, it has been documented that tumor cell killing by CTLs may lead to the release of tumor antigens that prime the generation of tumor antigen-specific T cells^8,47–49^. Subsequently, the trafficking of these CTLs to the tumor is important for killing tumor cells and re-invigorating the cancer immunity cycle^10,11^. In this regard, we found that targeting TCTP in tumor cells not only reversed tumor cell resistance to CTL-mediated apoptosis but also increased the number of infiltrated T cells in the tumors. Therefore, our study emphasized that blocking the TCTP immune-refractory signal axis may release the brakes that inhibit multiple steps in the anti-cancer immunity cycle, thereby improving immunotherapy efficacy.

In an effort to enhance anti-cancer immunity, therapeutic strategies, such as increasing tumor immunogenicity or T cell priming (tumor antigen or DC vaccines) and the cytotoxic capacity of CTLs by reversing the immunosuppressive tumor microenvironment (TME) (by depleting regulatory T cells (Tregs), myeloid-derived suppressor cells (MDSCs), or tumor-associated macrophages (TAM)) have been suggested for combination with T cell-mediated therapy^7^. However, an increased generation of tumor-specific T cells may not always lead to CTL trafficking to tumors. In addition, the inhibition of T cell exhaustion and immunosuppressive factors in the TME does not guarantee that the resistance of tumor cells to CTL-mediated killing will be conquered. Since our results provided a rationale that targeting TCTP to reinforce multiple steps in anti-cancer immune events, thereby overcoming immunotherapy resistance, we screened clinically available TCTP-targeting agents such as DHA, rapamycin, sertraline, and thioridazine. When the cells were treated with a sublethal dose of each agent, DHA was the most effective in impairing the resistance of TCTP^high^ tumor cells to CTL-mediated apoptosis (Fig. 6b). As evidenced by the combination score, the synergistic action of DHA and CTL was the best TCTP-targeting combination to kill tumor cells (Fig. 6c). It is notable that although the administration of other drugs (MEK or PI3K inhibitors, etc) targeting mechanisms underlying immunotherapy resistance has been stopped due to toxicity or yet clinical trials are still ongoing, DHA is a clinically available agent used to treat malaria^50^, and recently considered to be a therapeutic agent for multiple types of cancer^45,51^. Since the purpose of using DHA is to sensitize the TCTP^high^ tumor cells to T cell-mediated therapy, we treated low dose DHA (25 mg kg^−1^) for just two days before immunotherapy even other group treated DHA daily at 20 mg kg^−1^ to 133 mg kg^−1^ to reduced the tumor volume and weight^52,53^. Nevertheless, clinical application of DHA should be considered more carefully as T cell-mediated therapy requires long treatment, and TCTP is expressed in various type of non-tumor cells. Our results conceptually encourage the application of rational, mechanism-based combinations of TCTP-targeting drugs and immune-mediated therapy for the treatment of refractory tumor. Thus, finding new clinical applicable drugs that targeting TCTP, and developing new drugs to targeting TCTP with low side effect are needed to increase therapeutic efficacy and optimize outcome of cancer patient who refractory to ICB or ACT therapy.

In general, TCTP drives malignant phenotypes and the cross-resistance of tumor cells to chemo- and irradiation therapies by regulating oncogenic pathways such as the EGFR-AKT pathway^29,54^. Therefore, we believed that the TCTP-dependent oncogenic pathway might provide a route of resistance to T cell-mediated therapy. Here, we revealed that EGFR signaling mediates TCTP-induced resistance of tumor cells to CTL-mediated killing and decrease of T cell infiltration into the tumor by regulating MCL-1 and CXCL10. Knockdown of EGFR axis sensitized TCTP^high^ tumor cells to CTL-mediated killing, and increased T cell chemotaxis. Based on our molecular study, TCTP phosphorylation by PLK1 was crucial for binding with the Na, K ATPase α1 subunit, thereby activating the EGFR-AKT pathway. PLK1 has been considered an attractive target to treat multiple types of cancer^55,56^. Thus, its inhibitors, such as BI2536, BI6727, and GSK461364, have emerged promising anti-cancer agents and progressed to clinical trials^57^. In this regard, treatment with BI2536 reduced the immune-refractory properties of tumor cells (Supplementary Fig. 16b–d). Therefore, our findings reveal that pTCTP-EGFR-MCL-1/CXCL10 axis could be applied to reverse immune-refractory phenotypes, and suggest that blocking TCTP phosphorylation could be an additional promising strategy for combination with T cell-mediated therapy.

For the clinical application of treatment strategies combined with immunotherapy, the presence of biomarkers that predict clinical benefit is important for appropriately selecting the patients. Here, we found that the levels of *TPT1* within the tumor significantly correlated with the response to anti-PD-L1 therapy and survival in cancer patients, indicating that the expression status of *TPT1* may serve as a marker to predict the clinical outcomes of anti-PD-L1 therapy. In this regard, mRNA level of *TPT1* mRNA levels were negatively correlated with CD8^+^ T cell signature genes and positively correlated with anti-apoptotic process genes. It has been reported that the clinical efficacy of ICB therapy was associated with the intrinsic resistance of tumor cells to CTL-mediated apoptosis, and predictable by CD8^+^ T cell infiltration^10^. The expression of PD-L1 in tumor is a companion diagnostic biomarker to prescribe ICB, and PD-L1^+/^TIL^+^ tumors^58^. Given the evidence that targeting TCTP with DHA changed non-T cell-inflamed (TIL^−^) tumors into T cell-inflamed (TIL^+^) tumors (Fig. 7), treatment of PD-L1^+^/TIL^-^ or PD-L1^+^/TCTP^+^ patients with DHA may convert the tumor type to TIL^+^, thereby providing clinical benefits to ICB therapy. Taken together, our results indicate that TCTP could be both a valid target and a companion diagnostic marker providing a framework for patient selection to apply combined therapy of T cell-mediated therapy with TCTP-targeting agents.

Accumulating evidence has demonstrated that consistent and evolving reciprocal interaction between the anti-cancer immune system and tumor cells confers intrinsic- and/or extrinsic resistance to immunotherapy. In the immunoediting process, CTL-mediated immune selection drives the adaptation of tumor cells to host immune surveillance, thereby contributing to generating tumor cells with intrinsic resistance to CTL attack^12,14^. Conversely, tumor cells repress anti-tumor immunity by impairing TIL recruitment to the tumor and effector function, known as extrinsic-resistance^15,17,59^. Here, using ACT-refractory tumor models, we revealed the increase of TCTP expression during CTL-mediated therapy, which was likely due to the enrichment of TCTP^+^ tumor cells with immune-refractory phenotypes and survival advantage to T cell-mediated therapy (Fig. 4). This could be the evidence that supporting the enrichment of TCTP^high^ immune-refractory tumor cells may be the result of immune selection imposed by CTLs and acquired resistance during the T cell-mediated therapy. Notably, increase of TCTP expression is commonly observed in various type of immune-refractory tumor cells to tumor antigen-vaccination, antigen-specific T cell ACT, and anti-PD-L1 therapy (Supplementary Fig. 19). Since tumor antigen-specific CTLs are common key effector of these T cell-mediated therapies, the elevated TCTP expression seems to be reminiscent in multiple immune-refractory tumor cells. Therefore, targeting TCTP could be a potential combinational strategy enhancing the response to various type of T cell-mediated therapy. Furthermore, immune-refractory tumor cells trigger intrinsic- as well as extrinsic-resistance by conferring anti-apoptotic properties to the CTLs and reducing T cell recruitment to the tumor. This means that tumor cells evolved during T cell-mediated therapy not only evade the CTL-mediated lysis but also reversely restrict anti-cancer immunity via blocking T cell trafficking to the tumor. Taken together, our study revealed that the crucial dual role of the TCTP axis of tumor cells at the crossroads between tumor cells and the anti-cancer immunity system to potentiate resistance to T cell-mediated therapy.

In summary, we propose that TCTP^high^ tumor cells enriched by immune selection pressure drive immune-refractory phenotypes such as reduced T cell infiltration of the tumor and anti-apoptotic properties of the tumor. In this process, TCTP activates the EGFR-AKT signaling pathway by phosphorylation-dependent binding with Na, K ATPase α1. Furthermore, we demonstrated that the inhibition of TCTP with DHA induced tumor susceptibility to T cell-mediated therapy by enhancing anti-cancer immunity. In addition, the TCTP levels within the tumors significantly correlated with the clinical outcome of anti-PD-L1 therapy. Altogether, our findings indicate TCTP not only can be a companion marker it also can be a actionable target for combining TCTP-targeting agents to improve response to ACT and ICB therapy. Therefore, combining clinically ready agents targeting the TCTP axis with T cell-mediated therapy may be a promising strategy for better clinical outcomes.

## Methods

### Mice and cell lines

Female BALB/c and NOD/SCID mice 6 to 8 weeks old were purchased from Central Lab. Animal, Inc. (Seoul, Korea). For the in vivo experiments, 10 mice from each group were used. All mice were handled and maintained under the protocol approved by the Korea University Institutional Animal Care and Use Committee (KUIACUC-2017-0141). All animal procedures were performed in accordance with the recommendations for the proper use and care of laboratory animals.

CT26, A375, CaSki, 526Mel, MDA-MB-231, and HCT116 cells were obtained commercially from the American Type Culture Collection (ATCC, Manassas, VA, USA). All cell lines were purchased between 2010 and 2014 and tested for mycoplasma using a Mycoplasma Detection Kit (Thermo Fisher Scientific, San Jose, CA, USA). The identities of the cell lines were confirmed by short tandem repeat profiling by IDEXX Laboratories, Inc., and used within 6 months for testing. Pancreas PDC cell lines were generated by Suhwan Chang. The generation of the immuno-edited CaSki/P3 and MDA-MB-231/P3 cell lines was as described previously^19^. To generate the A375/TCTP cells, pMSCV-TCTP plasmids were first transfected along with viral packaging plasmid (VSVG and Gag-pol) into HEK293FT cells. After 3 days, the viral supernatant was filtered through a 0.45 μm filter and introduced into A375 cells. Then, the infected cells were selected with 1 μg ml^-1^ puromycin. For the generation of the A375/P3 tumor line, 1 × 10^6^ A375 cells were inoculated subcutaneously into NOD/SCID mice. After the initial tumor challenge, 2 × 10^6^ NY-ESO1-specific CD8+ T cells and 3000U of IL-2 (Novartis, Basel, Switzerland) were injected intravenously. After T cell adoptive transfer, the explanted tumor was expanded in vitro. This escape variant cell line was designated A375/P1 and injected into a new group of mice and selected by adoptive T-cell transfer again. This treatment regimen was repeated for three rounds. All cells were grown at 37 °C in a 5% CO_2_ humidified incubator chamber.

### Chemical reagents

The following chemical reagents were used in this study: BI2536 and cisplatin (Selleckchem, Houston, TX, USA). DHA, sertraline hydrochloride, and rapamycin (Sigma-Aldrich, USA), and thioridazine (Tocris, UK)

### DNA constructs

DNA fragments of the TCTP gene were generated with a PCR-based strategy from genomic DNA extracted from A549 cells using a primers for the *Bam*HI site, 5′-GGATCCATGATTATCTACCGGGAC-3′ and the *Xho*I site, 5′-CTCCAGTTAACATTTTTCCATTTCT-3′. The *Bam*HI and *Xho*I restriction fragments of the PCR product were subcloned into a pGEM-T vector (Promega, USA). TCTP fragments were subcloned into a pcDNA4/HisMax C vector containing a His-tag. pMSCV-*CXCL10* plasmids were purchased from Cosmogenetech (Seoul, KOR).

### Site-directed mutagenesis

To generate mutations in the TCTP phosphorylation sites, the QuikChange Site-directed Mutagenesis Kit (Stratagene, San Diego, CA, USA) was used according to the manufacturer’s instructions. The following primers were used: TCTP S46D forward 5′-GGTAACATTGATGACGACCTCATTGGTGGAAATGCCTCCGC-3′; reverse 5′-GCGGAGGCATTTCCACCAATGGAGGTCGTCATCAATGTTACC-3′, S46A forward 5′-CGAGGGCGAAGGTACCGAAGCAACAGTAATCACTGGTGTCG-3′; reverse 5′-CGACACCAGTGATTACTGTTGCTTCGGTACCTTCGCCCTCG-3′. The PCR thermal cycling conditions were 95 °C for 5 min; 18 cycles of 95 °C for 1 min, and 64 °C for 1 min, and 68 °C for 15 min. The PCR products were digested with *Dpn Ι* at 37 °C for 1 h and transformed into XL10-Gold ultracompetent bacterial cells. Mutations were confirmed through DNA sequencing.

### Real-time quantitative RT-PCR

Total RNA from the cells was purified using a RNeasy Micro kit (Qiagen, Valencia, CA, USA) and cDNA was synthesized by reverse transcriptase (RT) using an iScript cDNA synthesis kit (Bio-Rad, Hercules, CA, USA) according to the manufacturer’s recommended protocol. Real-time PCR was performed using iQ SYBR Green Super mix (Bio-Rad) with the specific primers on a CFX96 real-time PCR detection system. All experiments were performed in triplicate and the quantification cycle values were measured using Bio-Rad CFX96 Manager 3.0 software. Predesigned QPCR primers were purchased from Bioneer (South Korea); *TPT1* 5′-ATGACGAGCTGTTCTCCGAC-3′ (forward); 5′-AACACCGGTGACTACTGTGC-3′ (Reverse). Relative quantifications of the mRNA levels were performed using the comparative Ct method with β-ACTIN as the reference gene. Fold change was calculated relative to the expression level of mRNA in the control cells.

### siRNAs constructs

Synthetic small interfering RNAs *siGFP*, *siTPT1*, *siEGFR*, and *siMCL1* were purchased from Bioneer (South Korea), and had the following sequences: GFP, 5′-GCAUCAAGGUGAACUUCAA-3′ (sense), 5′-UUGAAGUUCACCUUGAUGC-3′ (antisense); mouse *TPT1* #1, 5′-GAAAUCACUCAAAGGCAAA-3′ (sense), 5′-UUUGCCUUUGAGUGAUUUC-3′ (antisense); mouse *TPT1* #2, 5′-CUGUUCUCCGACAUCUACA-3′ (sense), 5′-UGUAGAUGUCGGAGAACAG-3′ (antisense); mouse *TPT1* #3, 5′-AGCACAUCCUUGCUAAUUUTT-3′ (sense), 5′-AAAUUAGCAAGGAUGUGCUTA-3′ (antisense); human *TPT1*, 5′-GCAUGGUUGCUCUAUUGGA-3′ (sense), 5′-UCCAAUAGAGCAACCAUGC-3′ (antisense), mouse MCL-1, 5′–3′ (sense), 5′–3′ (antisense); human EGFR, 5′-AGGAAUUAAGAGAAGCAACAU-3′ (sense), 5′-AUGUUGCUUCUCUUAAUUCCU-3′ (antisense); mouse MCL-1, 5′-GGGCAGGAUUGUGACUCUUAUUUCU-3′ (sense), 5′-AGAAAUAAGAGUCACAAUCCUGCCC-3′ (antisense). siRNA was delivered into 6-well plates at a dosed of 200 pmol/well using Lipofectamine 2000 (Invitrogen, Carlsbad, CA, USA) in vitro. siRNA was delivered into mice after formulation with CNPs as described previously^12^ in vivo. Briefly, siRNA (1 μg μl^−1^) and tripolyphosphate (0.25% w/v) were combined in RGD-chitosan solution, and the mixture was incubated at 4 °C for 40 min. siRNA-loaded nanoparticles were purified by centrifugation and injected into the tail veins of tumor-bearing mice.

### Granzyme B apoptosis assays

GrB (Enzo Life Sciences, NY, USA) was delivered into cells by the BioPORTER QuikEasy Protein Delivery Kit (Sigma-Aldrich, St. Louis, MO, USA). Tumor cells (5 × 10^4^) were plated in 12-well plates and cultured overnight and 37 °C. The cells were washed and 200 ng of GrB with BioPORTER in Opti-MEM was added to each well. After incubation for 4-6 hours, the frequency of apoptotic cells was determined by staining with anti-active-caspase-3 antibody (BD Pharmingen, Cat 561011) and analyzed by flow cytometry FACSuite software.

### In vitro CTL assays

The tumor cells were harvested by trypsinization and washed once with DMEM (Thermo Fisher, USA) containing 0.1% fetal bovine (FBS), resuspended, and labeled in 0.1% DMEM with 10 µM CFSE for 10 minutes in a 37 °C incubator with 5% CO_2_. Then, the CFSE-labeled (526Mel, HCT116, CaSki, PDC110526) tumor cells were resuspended in 10 µM MART-1 peptide containing 1 ml of DMEM. In the case of A375 and CT26, the peptide-pulsing process was not needed. After peptide-pulsing for 1 h, the cells were incubated for 4 h with AH1-, MART-1- or the NY-ESO1-specific CD8+ T cell lines at an *E*/*T* ratio of 1:1. The frequency of apoptotic cells was analyzed by staining with anti-active caspase-3 antibody and performing flow cytometry. All analysis was performed using a Becton Dickinson FACSverse (BD Bioscience, USA).

### In vitro Transwell-based T cell chemotaxis assays

T cells were applied at 1 × 10^5^ cells/well to the upper wells of 3.0 µm 24-well cell culture inserts (Corning Lowell, MA, USA). The wells were filled with tumor cell-derived CM. After 4 h of incubation at 37 °C, the migrated T cells were collected from the bottom wells and counted.

### Cell viability assays

CT26 tumor cells were treated with indicated concentrations of cisplatin, DHA, rapamycin, sertraline, and thioridazine for 24 h. Cell viability was measured by the trypan blue exclusion assay. The data are expressed as the percentages of unstained cells compared to the control cells not exposed to the chemical reagents. The concentrations causing a 50% decrease in cell viability (IC_50_ values) were determined.

### Western blot analysis

Lysate extracted from a total of 5 × 10^5^ cells was used to perform western blots. Primary antibodies against TCTP (ab37506, Abcam), pTCTP(#5251, Cell signaling), pEGFR(Life technologies, 44784G), EGFR(#4267, Cell signaling), PLK1 (#4535, Cell signaling), MCL-1 (sc-819, Santa Cruz Biotechnology), CXCL10 (551215, BD Biosicences), β-actin (M177-3, MBL) were used. Western blotting was followed by incubation with the appropriate secondary antibodies conjugated to horseradish peroxidase, anti-rabbit IgG-HRP (Enzo, Cat ADI-SAB-300-J), and anti-mouse IgG-HRP (Enzo, Cat ADI-SAB-100-J). The immunoreactive bands were developed with the chemiluminescence ECL Detection system (GE Healthcare), and signals were detected using a luminescent image analyzer (LAS-4000 Mini).

### Immunohistochemistry (IHC)

Sections of formalin-fixed, paraffin-embedded (FFPE) CT26 P0 or P3 tumor tissues were deparaffinized, and prepared for antigen retrieval by citrate buffer (scytek) in microwave. Tissue sections were incubated with an anti-TCTP antibody (1:100–200; Abcam, ab13368) for 1 h 30 min at RT. After washing, sections were incubated with secondary antibodies conjugated to horseradish peroxidase (HRP) (ENZO, ADI-SAB-300) for 30 min at RT, and HRP was detected by diaminobenzidine (DAB; Dako) for 1 min. The analysis and quantification of IHC images were performed by using freeware ImageJ 1.53 g.

### Gene set used for signatures

Because KEGG pathways often include large numbers of genes with only loosely related functions, we constructed refined gene sets for the two core biologies called out in the text. Specifically:

- For the CD8+ T-effector signature genes, we used a previously published signature^10^.
- For the anti-apoptosis signature genes, we used genes within the negative regulation of apoptotic processes that were previously described^39^.

### Signature gene set score analysis

To investigate the clinical relevance in patients treated with anti-PD-L1 therapy, we used publicly available RNA sequencing data that have been deposited to European Genome-Phenome Archive under accession number EGAS00001002556. For gene expression analysis, the expression of each gene in a signature was first z-score-transformed. Then, a principal component analysis was performed, and principal component 1 was extracted by using R version 4.0.2 and R studio version 1.2.5033 to serve as a gene signature score as previously described^10^.

### In vivo tumor treatment experiments

To characterize the in vivo resistance to anti-PD-L1 conferred by TCTP, BALB/C mice were inoculated subcutaneously with 1 × 10^5^ CT26 tumor cells per mouse. Seven days following tumor challenge, *siGFP*- or *siTPT1*-loaded CNPs (5 μg/animal) was administered via intravenous injection for a day before anti-PD-L1 (BioXcell, NH, USA) (200 μg/mice) or isotype antibody control that was administrated via intraperitoneal injection every 3 days. To characterize the in vivo resistance to CTL killing conferred by TCTP, NOD/SCID mice were inoculated subcutaneously with 1 × 10^6^ A375 tumor cells per mouse. Seven days following tumor challenge, *siGFP*- or *siTPT1*-loaded CNPs (5 μg/animal) was administered via intravenous injection for a day before adoptive transfer with NY-ESO1-specific CTLs. This treatment protocol was repeated for 3 cycles. Mice were handled and monitored for tumor burden and survival under the protocol permitted by the Korea University Institutional Animal Care and Use Committee (KUIACUC-2017-0141). Tumor size was measured before the tumors were smaller than, or at about 10% of mice body weight, the maximal tumor size/burden permitted by KUIACUC. In some cases, this limit has been reached on the last day of tumor size measurement and the mice were immediately euthanized.

To analyze the immune cells in tumor, treated mice were euthanized on day 18 following tumor inoculation and the tumors were harvested. The tumors were filtered through a 70 μm cell strainer and washed with phosphate-buffered saline (PBS) buffer. The cell pellets were then incubated with red blood cell (RBC) lysis buffer for 2 min. The cell suspensions were stained for protein markers of interest. The staining antibodies used were anti-CD3 (1:100), anti-CD4 (1:100), anti-Foxp3 (1:50), anti-CD8 (1:100), anti-GrB, anti-active caspase 3. All antibodies were purchased from BD Biosciences.

### Statistics

All data shown are representative of at least three separate experiments. Statistical differences were calculated by either Student’s *t*-test (two-tailed, unpaired), one-way ANOVA, or two-way ANOVA using GraphPad Prism software version 7.0. Results with two-tailed *p* values of < 0.05 were considered statistically significant.

### Reporting summary

Further information on research design is available in the Nature Research Reporting Summary linked to this article.

## Supplementary information

Supplementary Information

Reporting Summary

## Data Availability

The RNA sequencing publicly available data used in this study are available in the European Genome-Phenome Archive under accession number EGAS00001002556. RNA-seq datasets from various cancer types are available in the Cancer Genome Atlas (TCGA) portal [https://tcga-data.nci.nih.gov/tcga/]. The gene ontology (GO) analysis which supported the findings of this study is publicly available online at [https://amp.pharm.mssm.edu/Enrichr/]. The gating strategy is provided in Supplementary Fig. 20. All raw images for the immunoblots are provided in Supplementary Fig. 21. The remaining data are available within the article and Supplementary Information. Source data are provided with this paper.

## Source data

Source data file
