# Supplementary material for: Targeting TCTP sensitizes tumor to T cell-mediated therapy by reversing immune-refractory phenotypes
Source: Nat Commun. 2022 Apr 19;13:2127. doi: 10.1038/s41467-022-29611-y (PMC9019109; doi:10.1038/s41467-022-29611-y)
Supplement: Supplementary file 1 — Supplementary Information [file 41467_2022_29611_MOESM1_ESM.docx]

**Supplemental Figures and Figure legends**

**Targeting TCTP sensitizes tumor to T cell-mediated therapy by reversing immune-refractory phenotypes**

Hyo-Jung Lee, et al.

**Supplemental Figures and Figure legends**

**
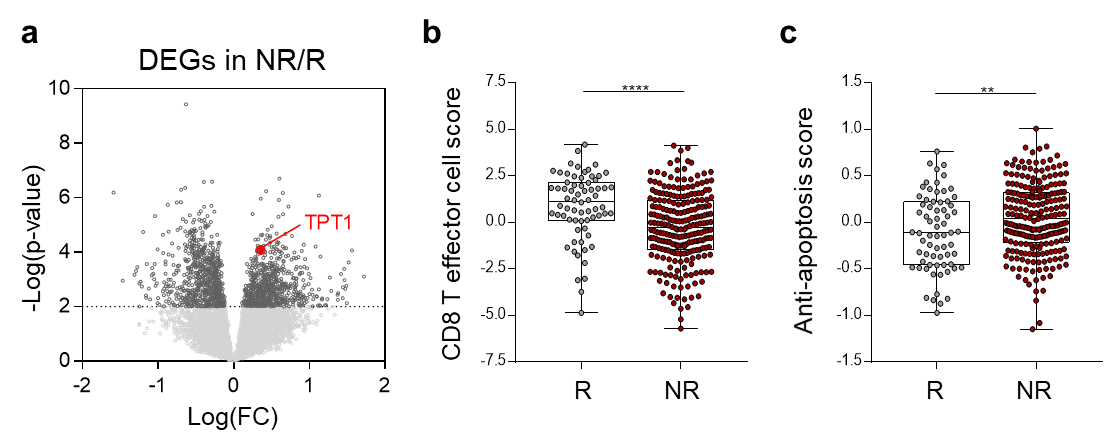
**

**Supplemental figure 1. Differentially expressed gene (DEGs) analysis, and signature gene scoring in responders and non-responders to anti-PD-L1 therapy.**

**a** Volcano plot shows the DEGs between the non-responders and responders to PD-L1 blockade therapy. The dot colors indicate significantly (deep gray) or non-significantly (light gray) altered genes in above or below the horizontal dashed line at *p* = 0.01. The red dot indicates the *TPT1* gene. The *p*-values were determined by a two-tailed test. **b** CD8^+^ T cell signature scores in responders (R, *n* = 60), and non-responders (NR, *n* = 230) to PD-L1 blockade therapy. **c** Anti-apoptosis scores in responders (R, *n* = 60), and non-responders (NR, *n* = 230) to PD-L1 blockade therapy. In the dot plots, the center lines indicate the medians, and the ends of the whiskers indicate the maximum and minimum values. The *p*-value by two-tailed *t* test **b** and **c** are indicated. Data represent the mean ± SD. Source data are provided as a Source Data file.


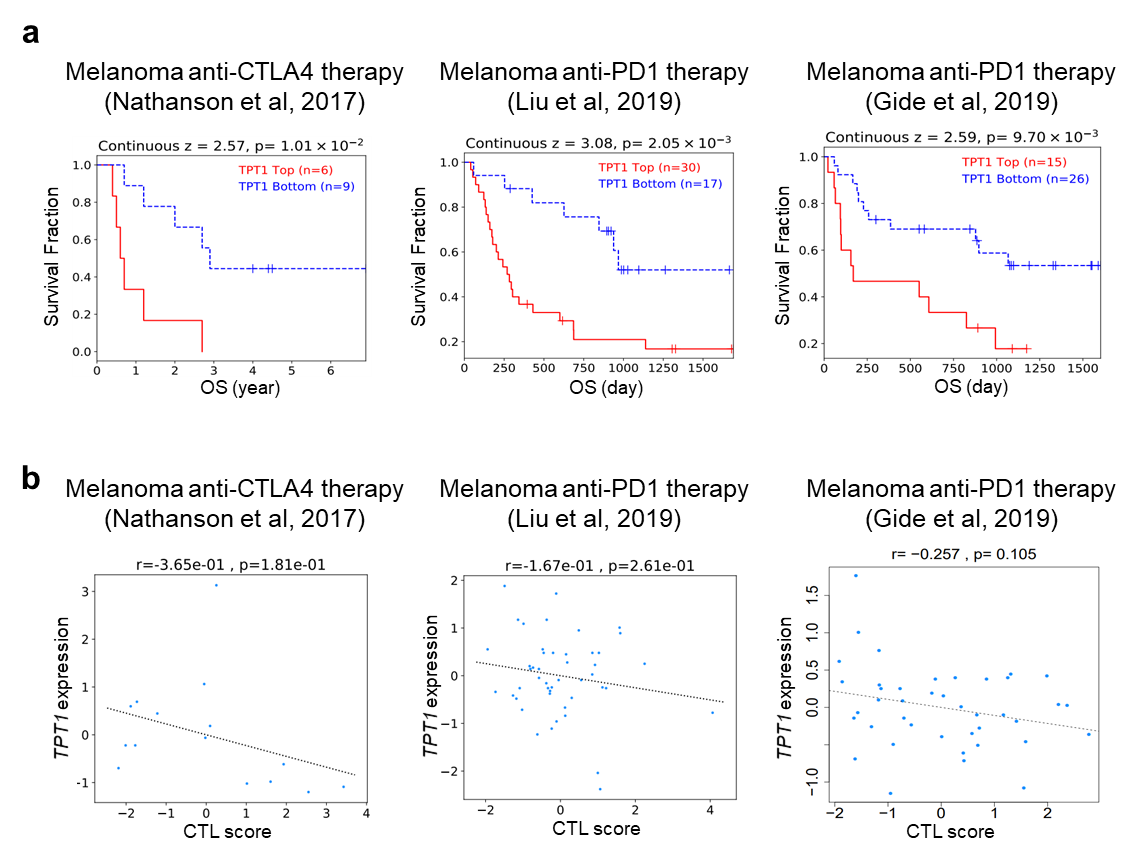


**Supplemental figure 2. Overall survival and correlation between CTL score and *TPT1* expression in cancer patients received ICB therapy.**

**a** Kaplan–Meier plots of indicated patient cohorts^1-3^ received ICB therapy with top half and bottom half *TPT1* expression levels was analyzed by using Tumor Immune Dysfunction and Exclusion (TIDE) website. The number of patients are indicated in graph legend, respectively. The *p-*values were computed through the two-sided Wald test in the Cox-PH regression. **b** Correlation between CTL score and *TPT1* expression was analyzed by using TIDE. Pearson correlation with cytotoxic T lymphocyte level were calculated. Source data are provided as a Source Data file.


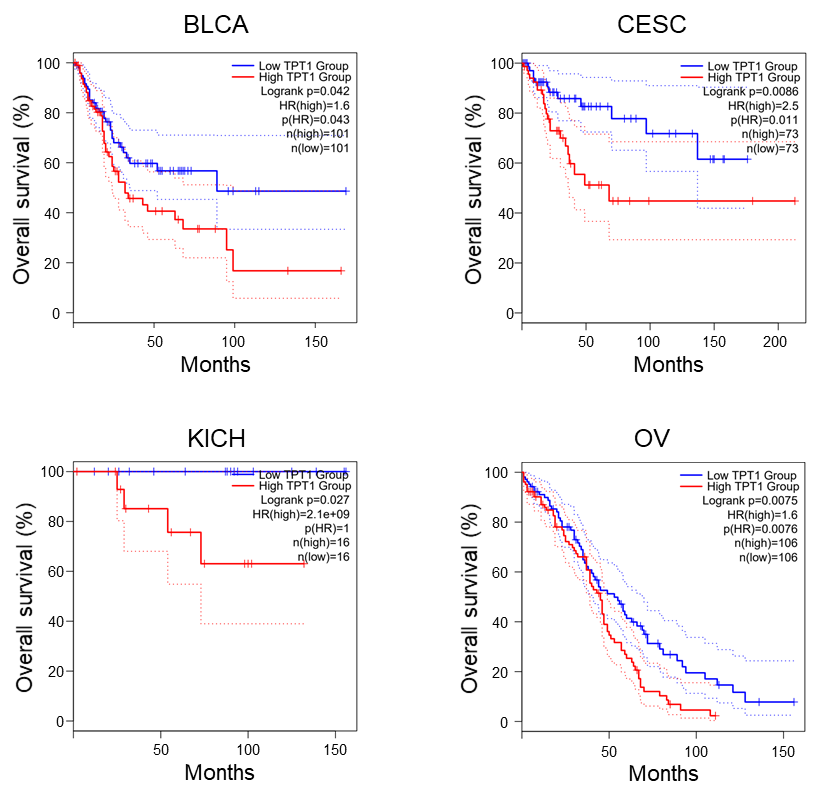


**Supplemental figure 3. Overall survival for various type of cancer patients according to *TPT1* expression.**

Overall survival of indicated various type of *TPT1*^low^ and *TPT1*^high^ patients from The Cancer Genome Atlas (TCGA) data organized in the Gene Expression Profiling Interactive Analysis (GEPIA2) website. The number of patients are indicated in graph legend, respectively. The logrank *p*-values were indicated in each plot. Source data are provided as a Source Data file.


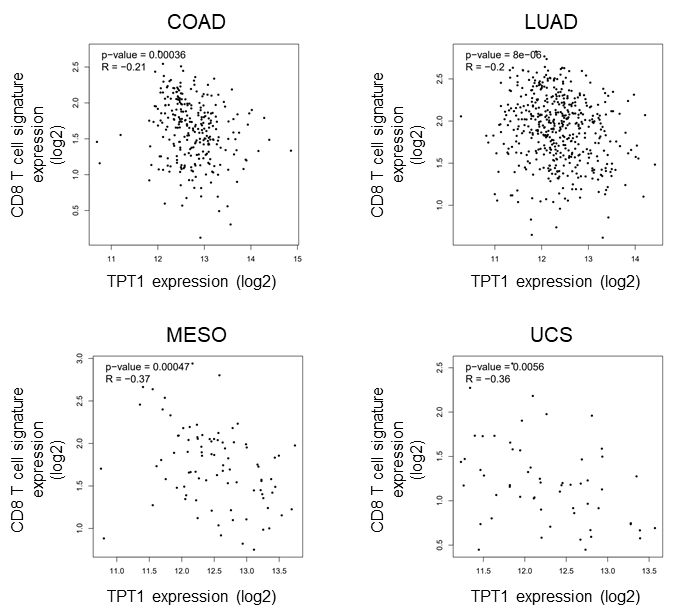


**Supplemental figure 4. CD8 T cell signature score is negatively correlated with *TPT1* expression in various type of cancer patients.**

Correlation between *TPT1* expression and CD8+ T cell signature genes expression in indicated various type of cancer patients (COAD (*n* = 275), LUAD (*n* = 483), MESO (*n* = 87), and UCS (*n* = 57)) from The Cancer Genome Atlas (TCGA) data organized in the Gene Expression Profiling Interactive Analysis (GEPIA2) website. Correlation analysis was performed using Spearman correlation coefficient. Source data are provided as a Source Data file.

**
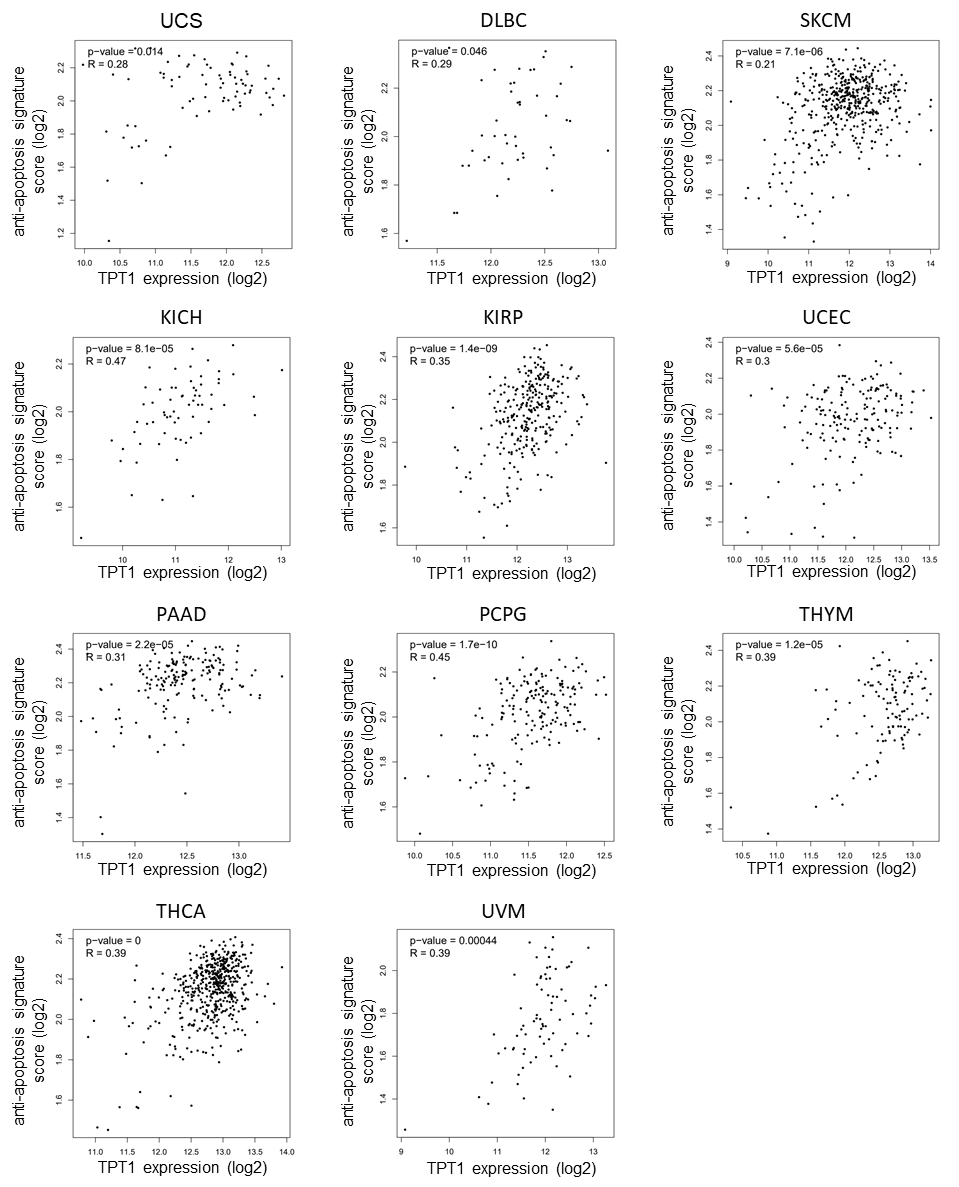
**

**Supplemental figure 5. Anti-apoptosis signature score is positively correlated with *TPT1* expression in various type of cancer patients.**

Correlation between *TPT1* expression and anti-apoptosis signature genes expression in indicated various type of cancer patients (UCS (*n* = 57), DLBC (*n* = 47), SKCM (*n* = 461), KICH (*n* = 66), KIRP (*n* = 286), UCEC (*n* = 174), PAAD (*n* = 179), PCPG (*n* = 182), THYM (*n* = 118), THCA (*n* = 512), UVM (*n* = 79) from The Cancer Genome Atlas (TCGA) data organized in the Gene Expression Profiling Interactive Analysis (GEPIA2) website. Correlation analysis was performed using Spearman correlation coefficient. Source data are provided as a Source Data file.

**
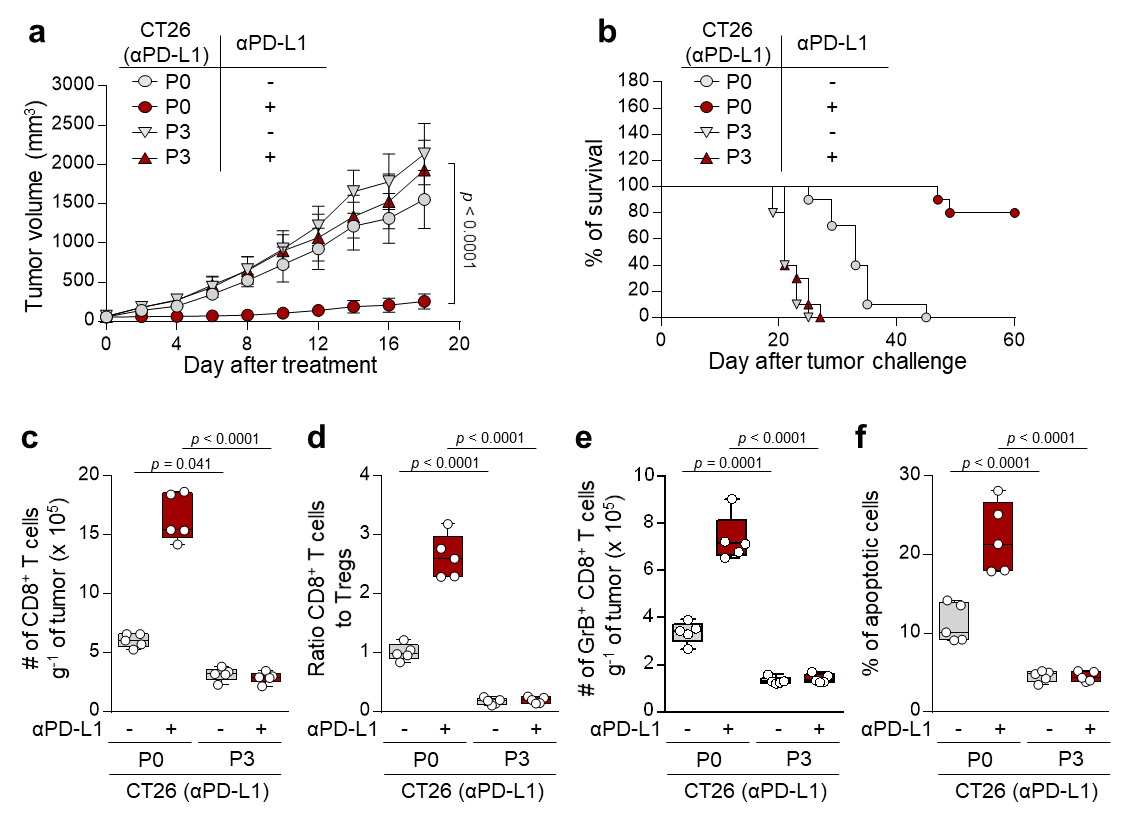
**

**Supplemental figure 6. *In vivo* characterization of ICB-refractory CT26 P3 model to anti-PD-L1 therapy. a-f** CT26 tumor-bearing mice treated or not treated with PD-L1 antibody. **a** Tumor growth and **b** survival of mice inoculated with CT26 P0 or P3 cells treated with or without PD-L1 antibody. **c** Flow cytometry profiles of tumor-infiltrating CD8+ T cells. **d** Tumor-infiltrating CD8^+^ T cell to CD4^+^, Foxp3^+^ Treg cell ratio. **e** The absolute number of granzyme B^+^ to tumor-infiltrating CD8^+^ T cells. **f** The frequency of apoptotic cells in the tumors treated with the indicated reagents. For the *in vivo* experiments, 10 mice from each group were used, and randomly selected 5 samples were analyzed **c-f.** The *p*-values by two-way ANOVA **a**, one-way ANOVA **c**-**f**, and the log-rank (Mantel-Cox) test **b** are indicated. The data represent the mean ± SD. Source data are provided as a Source data file.


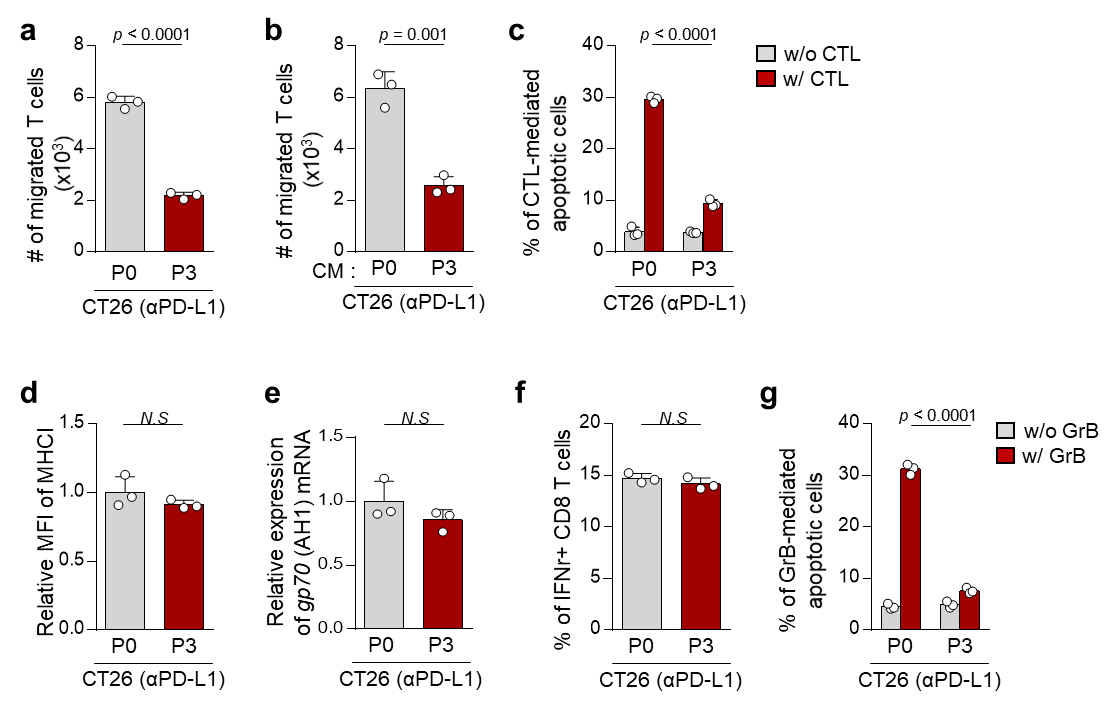


**Supplemental figure 7. Characterization of CT26 P3 model *in vitro*.**

**a** CT26 P0 or P3 cells were cultured in the lower chamber, and AH1-specific T cells were plated in the upper chamber. The T cells that migrated into the lower chamber were counted. **b** T cell chemotaxis assays were performed using CT26 P0 or CT26 P3 cell-derived CM in the lower chamber, and plating CD8^+^ T cells in the upper chamber. The T cells that migrated into the lower chamber media were counted. **c** CFSE-labeled tumor cells were incubated with or without tumor-specific CTLs and the frequency of CFSE^+^ apoptotic tumor cells was determined by flow cytometric analysis of active-caspase-3. **d** MHC class I expression on CT26 P0 and P3 cells was measured by flow cytometry. **e** The Relative mRNA expression of *gp70* (AH1) was measured by qRT-PCR. **f** CT26 P0 or P3 cells were incubated with AH1-specific T cells at a 1:1 effector : target ratio for 16 hours. The cells were then stained for surface CD8 and intracellular IFNγ to detect CTL activation. **g** Flow cytometry analysis of the frequency of apoptotic (active caspase3^+^) cells in the cells after intracellular delivery of granzyme B for 6 hours. All experiments were performed in triplicate. The *p*-values by two-tailed Student’s *t* test **a, b, d-f,** and two-way ANOVA **c**, **g** are indicated. *N.S*, not significant. The data represent the mean ± SD. Source data are provided as a Source data file.


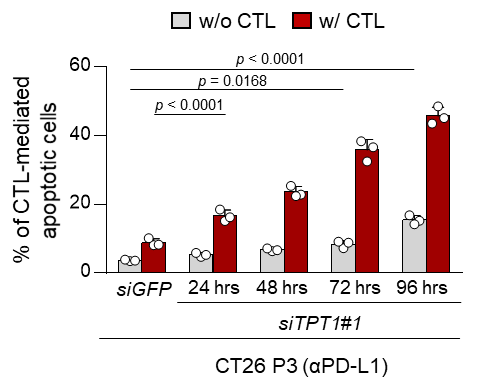


**Supplemental figure 8. TCTP-dependent apoptosis of CT26 P3 tumor cells with or without CTLs.**

CT26 P3 cells were treated with the *TPT1*-targeting siRNA from 24 to 96 hours. CT26 P3 tumor cells were labeled with CFSE and incubated with tumor-specific CTLs at 1:1 ratios. The frequency of CFSE^+^ apoptotic tumor cells was determined by flow cytometric analysis of active-caspase-3. All experiments were performed in triplicate. The *p*-values by two-way ANOVA are indicated. The data represent the mean ± SD. Source data are provided as a Source data file.


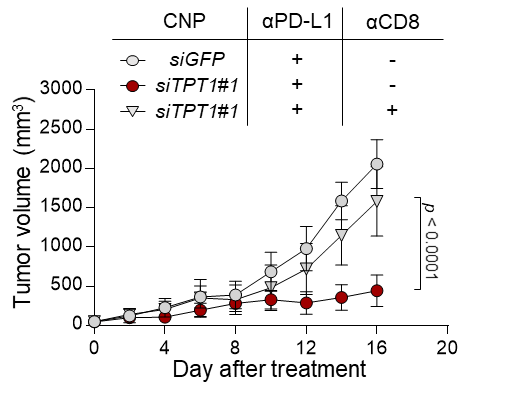


**Supplemental figure 9. Combined therapy with *siTPT1*-loaded CNPs and anti-PD-L1 therapy inhibits tumor growth via CD8^+^ T cells.**

Tumor growth of mice inoculated with P3 cells treated with or without PD-L1 antibody and/or CD8 depletion antibody. For the *in vivo* experiments, 10 mice from each group were used. The *p*-value by two-way ANOVA is indicated. Data represent the mean ± SD. Source data are provided as a Source data file.


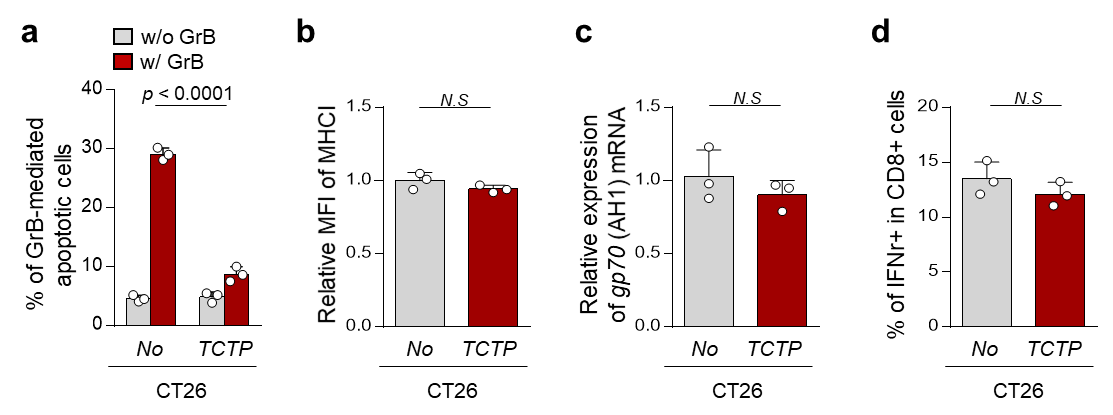


**Supplemental figure 10. MHC class I expression on the surface of cancer cells and CTL activation capacity by cancer cells are unaffected by TCTP expression.**

**a** Flow cytometry analysis of the frequency of apoptotic (active caspase 3^+^) cells in the total cells after intracellular delivery of granzyme B for 6 hours. **b** The MHC class I expression on CT26 P0 and P3 cells was measured by flow cytometry. **c** The Relative mRNA expression levels of *gp70* (AH1) was analyzed by qRT-PCR. **d** AH1-specific T cells were incubated with tumor cells at a 1:1 effector : target ratio for 16 hours. The cells were then stained for surface CD8 and intracellular IFNγ to detect CTL activation. All experiments were performed in triplicate. The *p*-values by two-way ANOVA **a**, two-tailed Student’s *t* test **b-d** are indicated. The data represent the mean ± SD. *N.S*, not significant. Source data are provided as a Source data file.


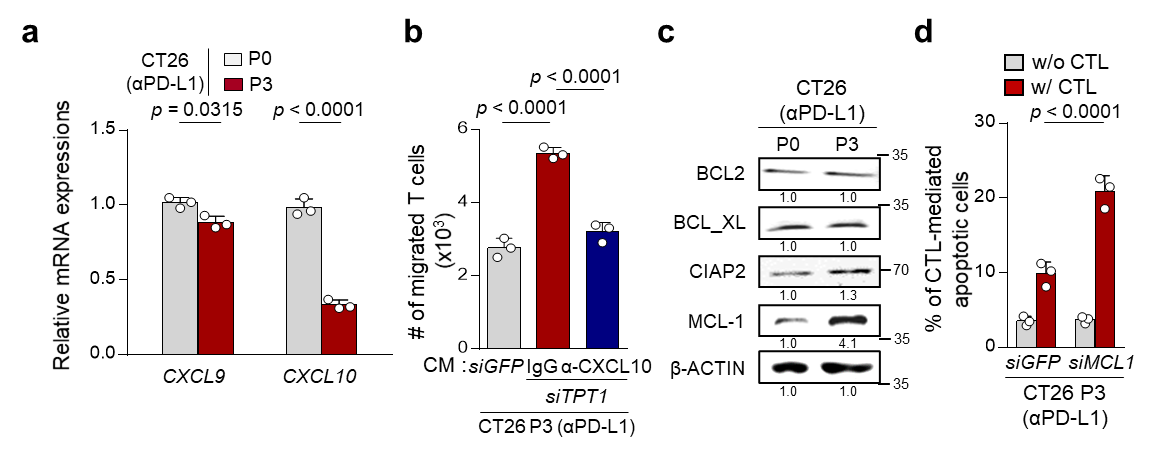


**Supplemental figure 11. CXCL10 and MCL-1 are key mediators in TCTP-induced immune-refractory phenotypes.**

**a** The relative mRNA expression of *CXCL9* and *CXCL10* was analyzed by qRT-PCR. **b** T cell chemotaxis assays were performed using the indicated tumor cell-derived CM. **c** The protein expression levels of BCL2, BCL_XL, CIAP2, MCL-1, and β-ACTIN were analyzed by Western blots. **d** Apoptotic tumor cells after incubation with or without CTLs were determined by flow cytometric analysis of active-caspase 3. **a, b,** and **c** experiments were performed in triplicate. The numbers below the blot images indicate the expression measured as fold-change **c**. The experiments were performed in triplicate. The *p*-values by two-way ANOVA **a, d**, and one-way ANOVA **b** are indicated. The data represent the mean ± SD. Source data are provided as a Source data file.

**
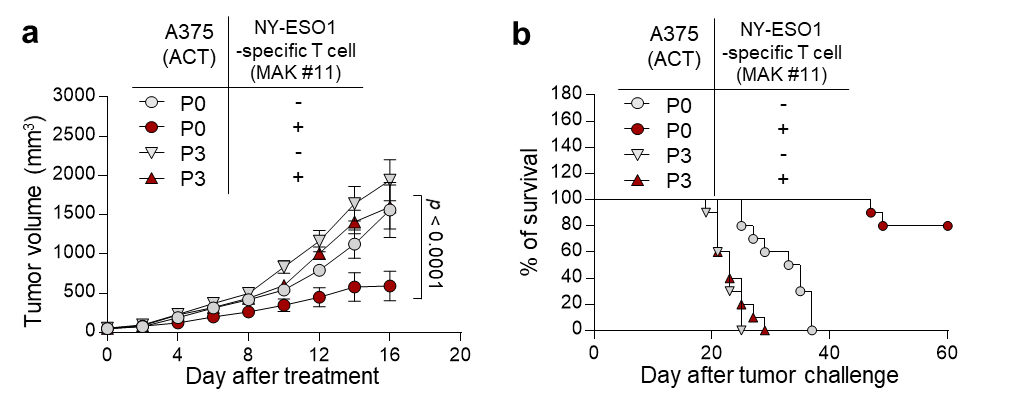
**

**Supplemental figure 12. Immune-refractory phenotypes of the A375 P3 tumor model *in vivo*.**

**a, b** Tumor-bearing mice with or without NY-ESO1-specific T cells adoptive transfer treatment. **a** Tumor growth and **b** survival of mice inoculated with A375 P0 or P3 cells treated with or without NY-ESO1-specific T cells adoptive transfer treatment. For the *in vivo* experiments, 10 mice from each group were used. The *p*-value by two-way ANOVA **a** and the log-rank (Mantel-Cox) test **b** are indicated. The data represent the mean ± SD. Source data are provided as a Source data file.


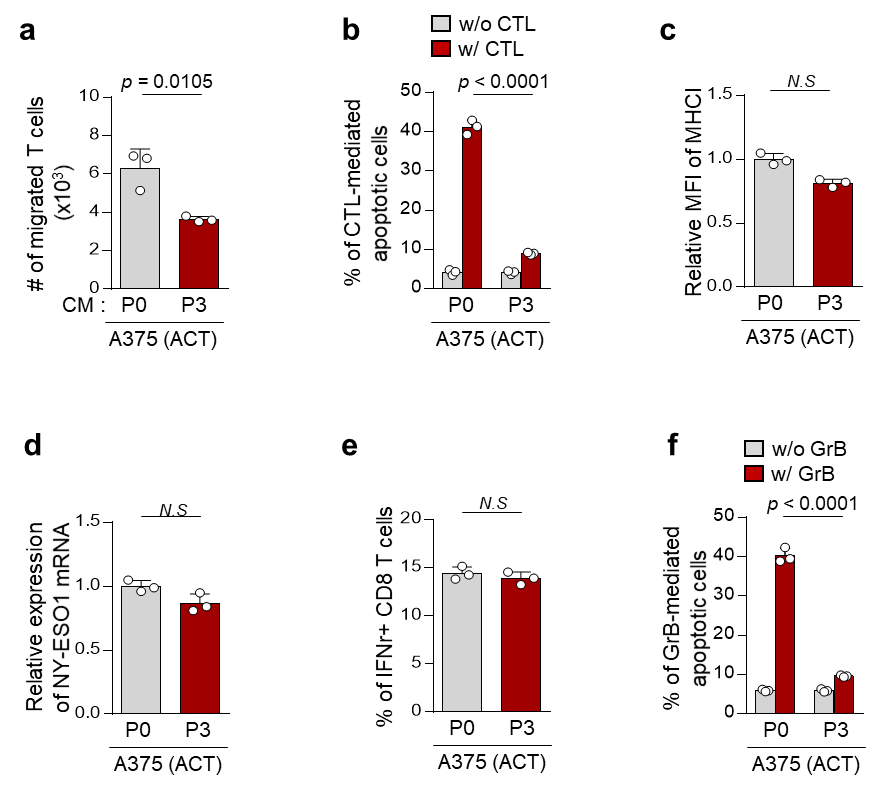


**Supplemental figure 13. *In vitro* characterization of ACT-refractory A375 P3 model.**

**a** T cell chemotaxis assays were performed using A375 P0 or A375 P3 cells-derived CM in the lower chamber, and plating CD8^+^ T cells in the upper chamber. The T cells that migrated into the lower chamber media were counted. **b** CFSE-labeled tumor cells were incubated with or without NY-ESO-1-specific CTLs and the frequency of CFSE^+^ apoptotic tumor cells was determined by flow cytometric analysis of active-caspase-3. **c** MHC class I expression on A375 P0 and P3 cells was measured by flow cytometry. **d** The Relative mRNA expression levels of *NY-ESO1* was analyzed by qRT-PCR. **e** A375 P0 or P3 cells were incubated with NY-ESO-1-specific T cells at a 1:1 effector: target ratio for 16 hours. The cells were then stained for surface CD8 and intracellular IFNγ to detect CTL activation. **f** Flow cytometry analysis of the frequency of apoptotic (active caspase 3^+^) cells in the cells after intracellular delivery of granzyme B for 6 hours. All experiments were performed in triplicate. The *p*-values by two-tailed Student’s *t* test **a, c, d, e,** and two-way ANOVA **b, f** are indicated. *N.S*, not significant. The data represent the mean ± SD. Source data are provided as a Source data file.


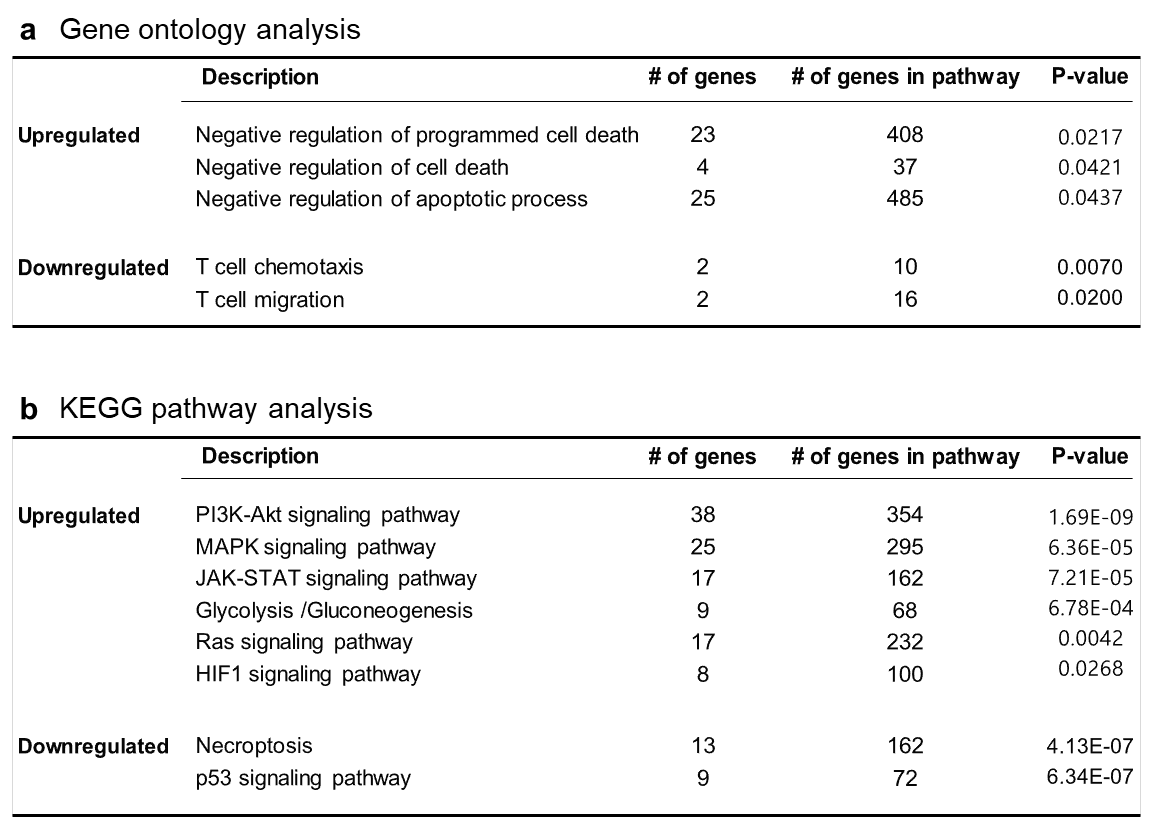


**Supplemental figure 14. Gene ontology and signaling pathway analysis using increased or decreased gene set of *TPT1*^high^ patients compared to *TPT1*^low^ patients.**

**a** Gene ontology enrichment analysis were performed using the up- and downregulated genes in *TPT1*^high^ patients (n = 147) compared to *TPT1*^low^ patients (n = 201). The cutoff values for the expression level of *TPT1* (*TPT1*^high^ > average; *TPT1*^low^ < average). **b** KEGG pathway analysis represented by the genes up- and downregulated in *TPT1*^high^ patients compared to *TPT1*^low^ patients. The *p*-values were calculated by Fisher exact test. Source data are provided as a Source Data file.


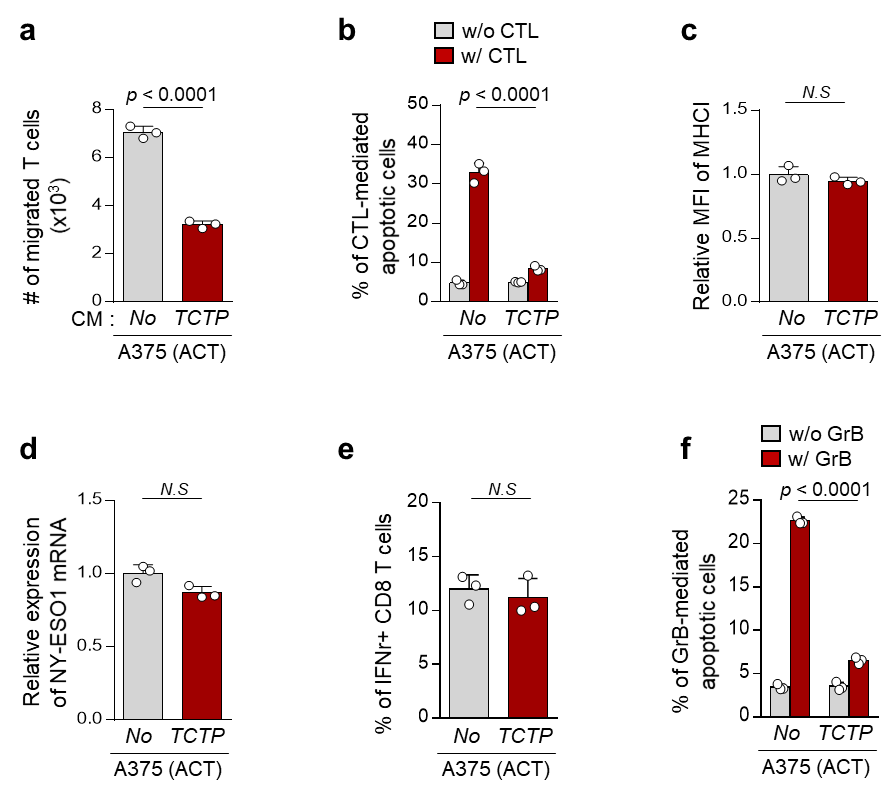


**Supplemental figure 15. MHC class I expression on the surface of cancer cells and CTL activation capacity by cancer cells are unaffected by TCTP expression.**

**a** T cell chemotaxis assays were performed using CM derived A375 No or A375 TCTP cells in the lower chamber, and CD8^+^ T cells in the upper chamber. The T cells that migrated into the lower chamber media were counted. **b** Tumor cells were labeled with CFSE and incubated with or without NY-ESO-1-specific CTLs. The frequency of CFSE^+^ apoptotic tumor cells was determined by flow cytometric analysis of active-caspase-3. **c** MHC class I expression on indicated tumor cells was measured by flow cytometry. **d** The relative mRNA expression levels of *NY-ESO1* was analyzed by qRT-PCR. **e** A375 No or TCTP cells were incubated with NY-ESO-1-specific T cells at a 1:1 effector: target ratio for 16 hours. The percentage of CD8+ and intracellular IFNγ+ T cells were analyzed by flow cytometry. **f** Flow cytometry analysis of the frequency of apoptotic (active caspase 3^+^) cells in the cells after intracellular delivery of granzyme B for 6 hours. All experiments were performed in triplicate. The *p*-values by two-tailed Student’s *t* test **a, c, d, e,** and two-way ANOVA **b, f** are indicated. *N.S*, not significant. The data represent the mean ± SD. Source data are provided as a Source data file.


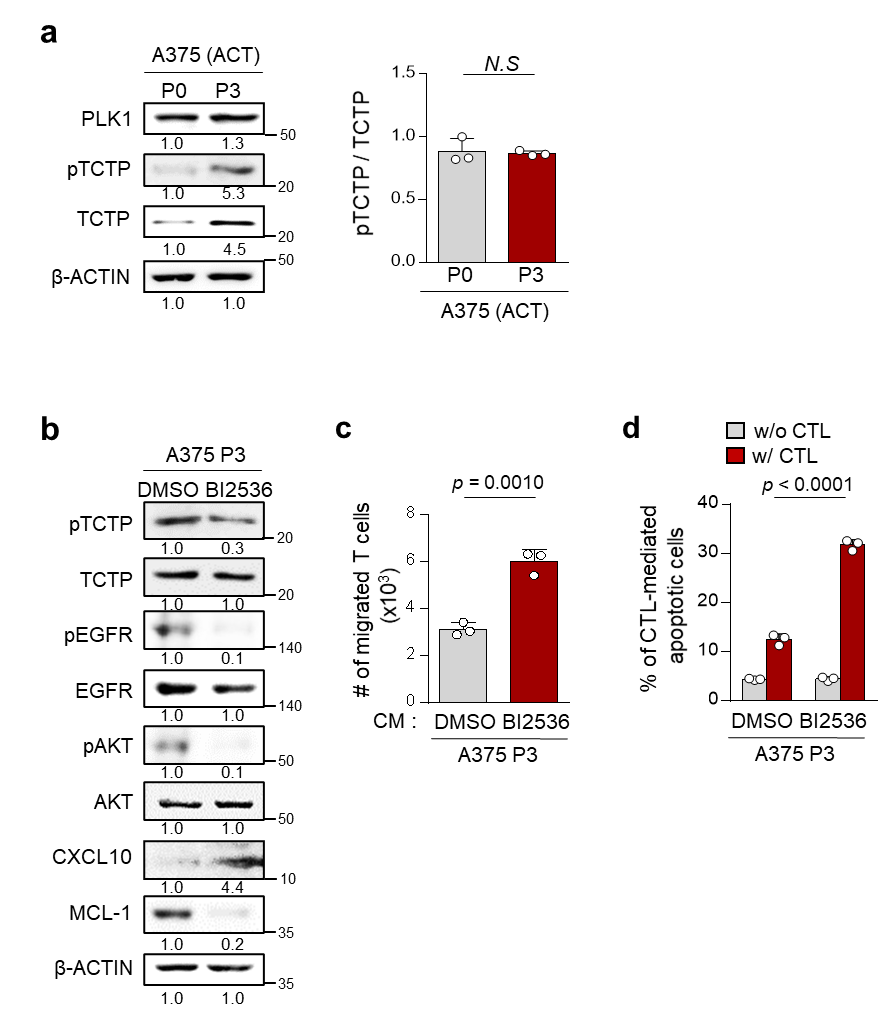


**Supplemental figure 16. Blocking TCTP phosphorylation reversed immune-refractory phenotypes of A375 P3 cells.**

**a** The protein levels of pTCTP, TCTP, PLK1, and β-ACTIN were demonstrated by Western blot analysis. The graphs represent quantification for ratio of pTCTP and total TCTP level. **b** The expression of pTCTP, TCTP, pEGFR, EGFR, pAKT, AKT, MCL-1, CXCL10, and β-ACTIN protein were analyzed by Western blots. **c** T cell chemotaxis assays were performed using dimethyl sulfoxide (DMSO)- or BI2536-treated A375 P3 cell-derived CM in the lower chamber, and plating NY-ESO-1-specific T cells in the upper chamber. The T cells that migrated into the lower chamber media were counted. **d** A375 P3 cells were treated with DMSO or BI2536 for 24 hours. Then, the tumor cells were labeled with CFSE and incubated with NY-ESO-1-specific CTLs. The frequency of CFSE^+^ apoptotic tumor cells was determined by flow cytometric analysis of active-caspase-3. The numbers below the blot images indicate the expression as fold-change **a** and **b**. The experiments were performed in triplicate. The *p*-values by two-tailed Student’s *t* test **a, c,** and two-way ANOVA **d** are indicated. *N.S*, not significant. The data represent the mean ± SD. Source data are provided as a Source data file.


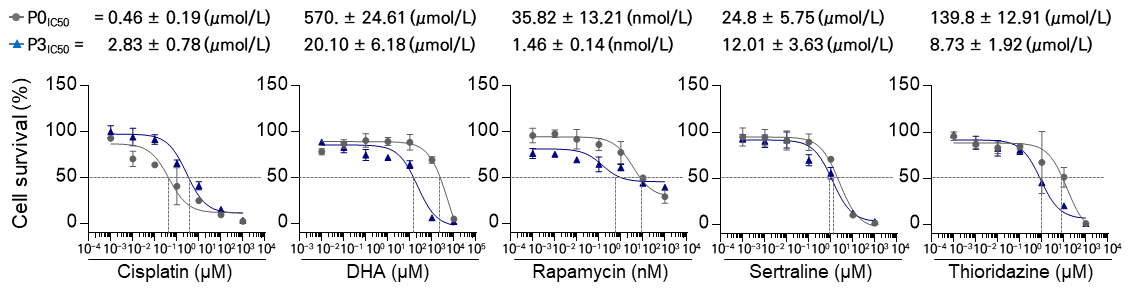


**Supplemental figure 17. Sensitivity of CT26 P0 and P3 cells to cisplatin or various TCTP-targeting agents.**

CT26 P0 and P3 cells were treated with the indicated concentrations of cisplatin, DHA, rapamycin, sertraline, and thioridazine for 24 hours. The cells viability was measured by trypan blue exclusion assay, and the concentrations causing a 50% decrease in cell viability (IC_50_ values) were determined. All experiments were performed in triplicate. The data represent the mean ± SD. Source data are provided as a Source data file.

**
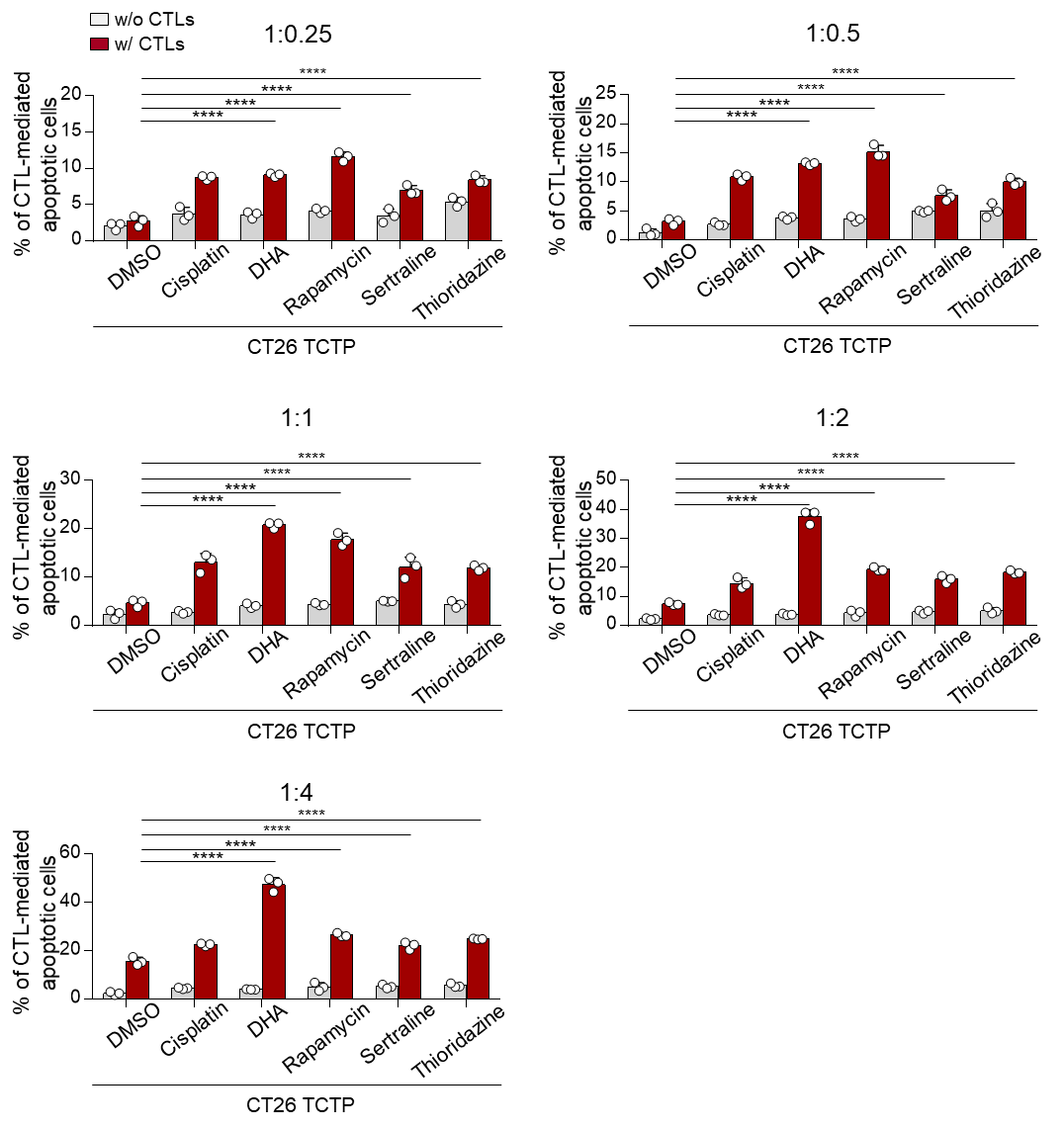
**

**Supplemental figure 18. Combining each TCTP targeting agent with CTLs enhanced CTL-mediated killing of tumor cells**

CT26 TCTP cells were treated with the indicated concentrations of cisplatin, DHA, rapamycin, sertraline, and thioridazine for 24 hours. Then, the CT26 TCTP cells were labeled with CFSE and incubated with tumor-specific CTLs at the indicated effector to target ratios. The frequency of CFSE^+^ apoptotic tumor cells was determined by flow cytometric analysis of active-caspase-3. All experiments were performed in triplicate. **p* < 0.05,***p* < 0.01, ****p* < 0.001, and *****p* < 0.0001. The *p*-values by two-way ANOVA are indicated. The data represent the mean ± SD. Source data are provided as a Source data file.

**
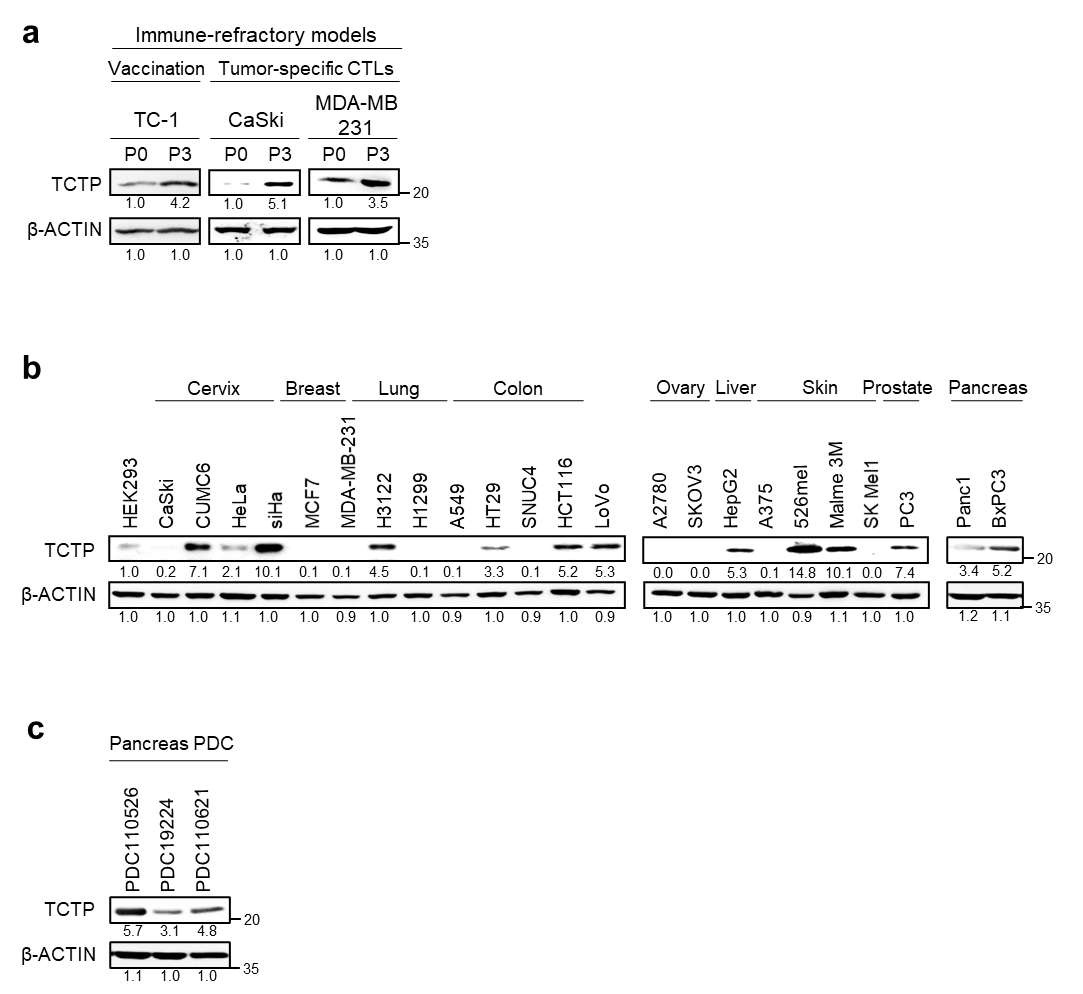
**

**Supplemental figure 19. Expression of TCTP in various types of immune-resistant tumor models and human cancer cell lines**

**a** The expression of TCTP in various type of immune-refractory tumor models was measured by Western blot analysis. **b** The expression of TCTP protein in various types of human cancer cell lines was measured by Western blot analysis. **c** The protein level of TCTP in pancreas PDC lines was analyzed by Western blot analysis The numbers below the blot images indicate the expression as fold-change. The experiments were performed in triplicate. Source data are provided as a Source data file.

**
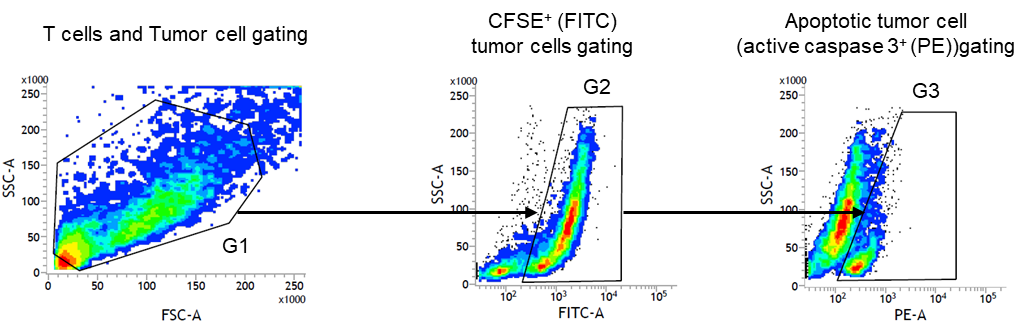
**

**Supplemental figure 20. Gating strategy for identification of apoptotic tumor cells by CTL-mediated killing.**

Representative flow cytometry analysis showing the gating strategy. T cells and tumor cells population was initially gated on the basis of the forward side scatter characteristics and debris were excepted. After then, CFSE^+^ tumor cells were gated in the plot CFSE (FITC). Finally, the CFSE^+^ and active caspase-3^+^ population was measured in the active caspase-3 (PE).

**Supplemental figure 21. Uncropped western blot images**


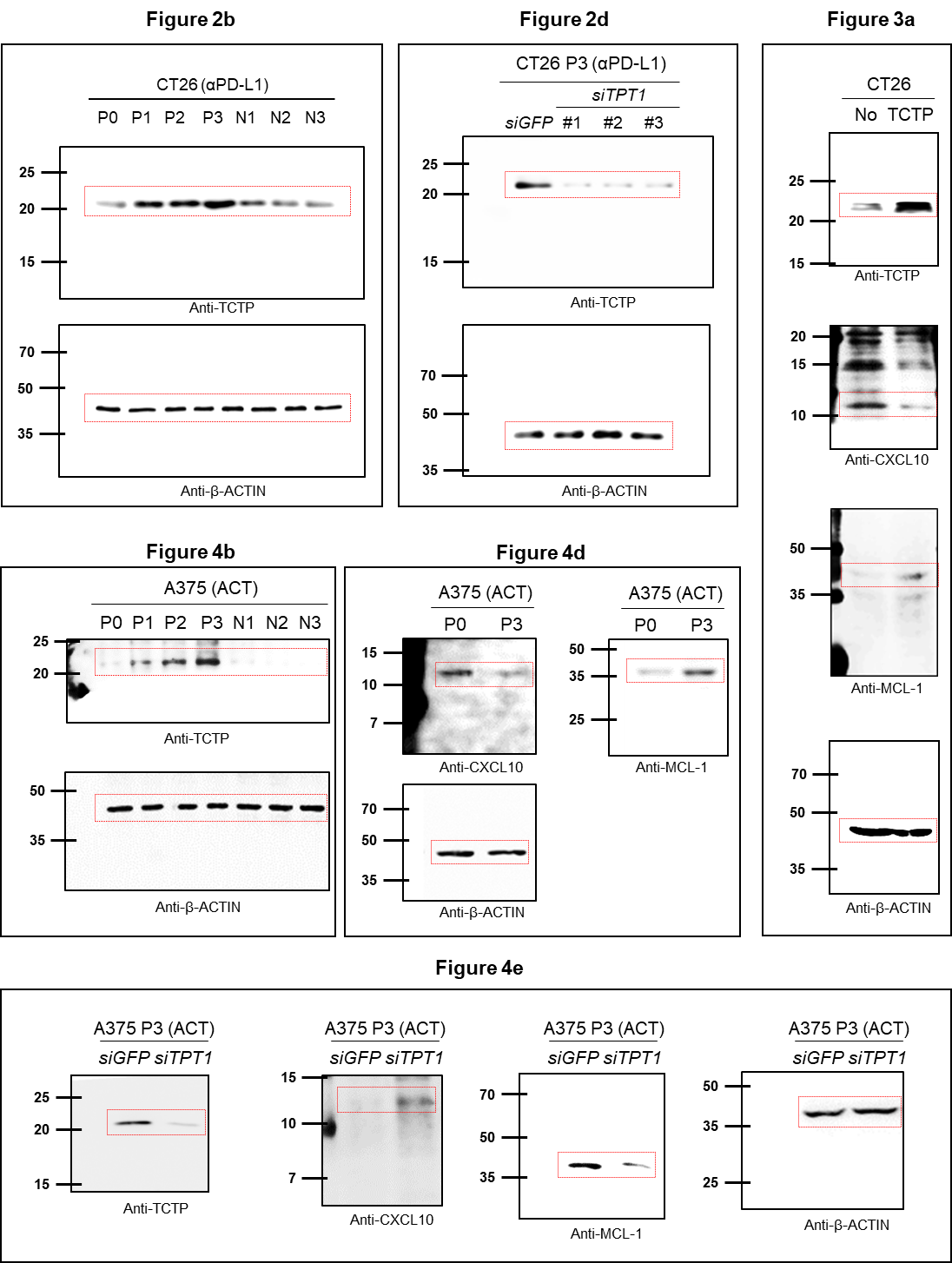


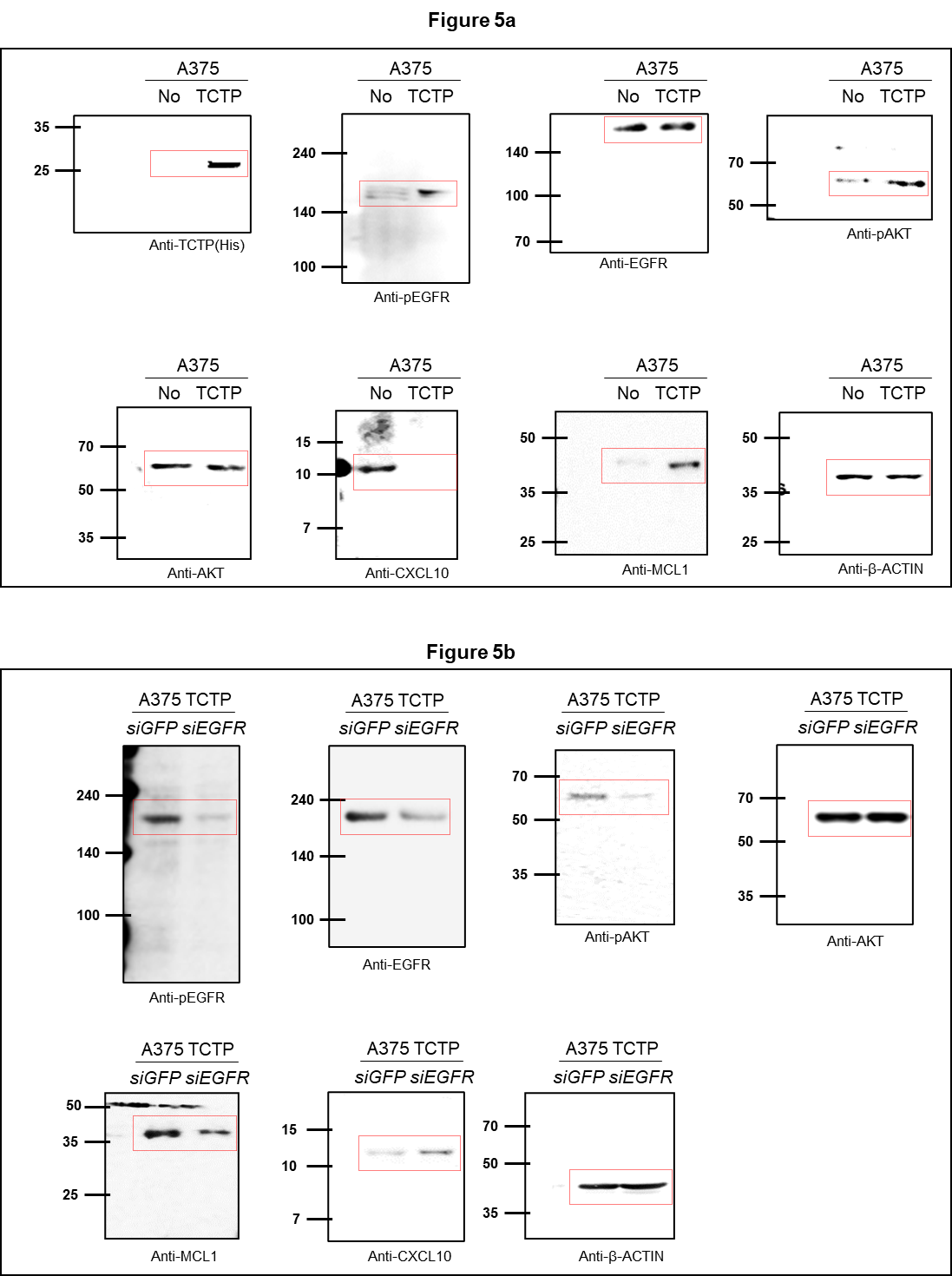


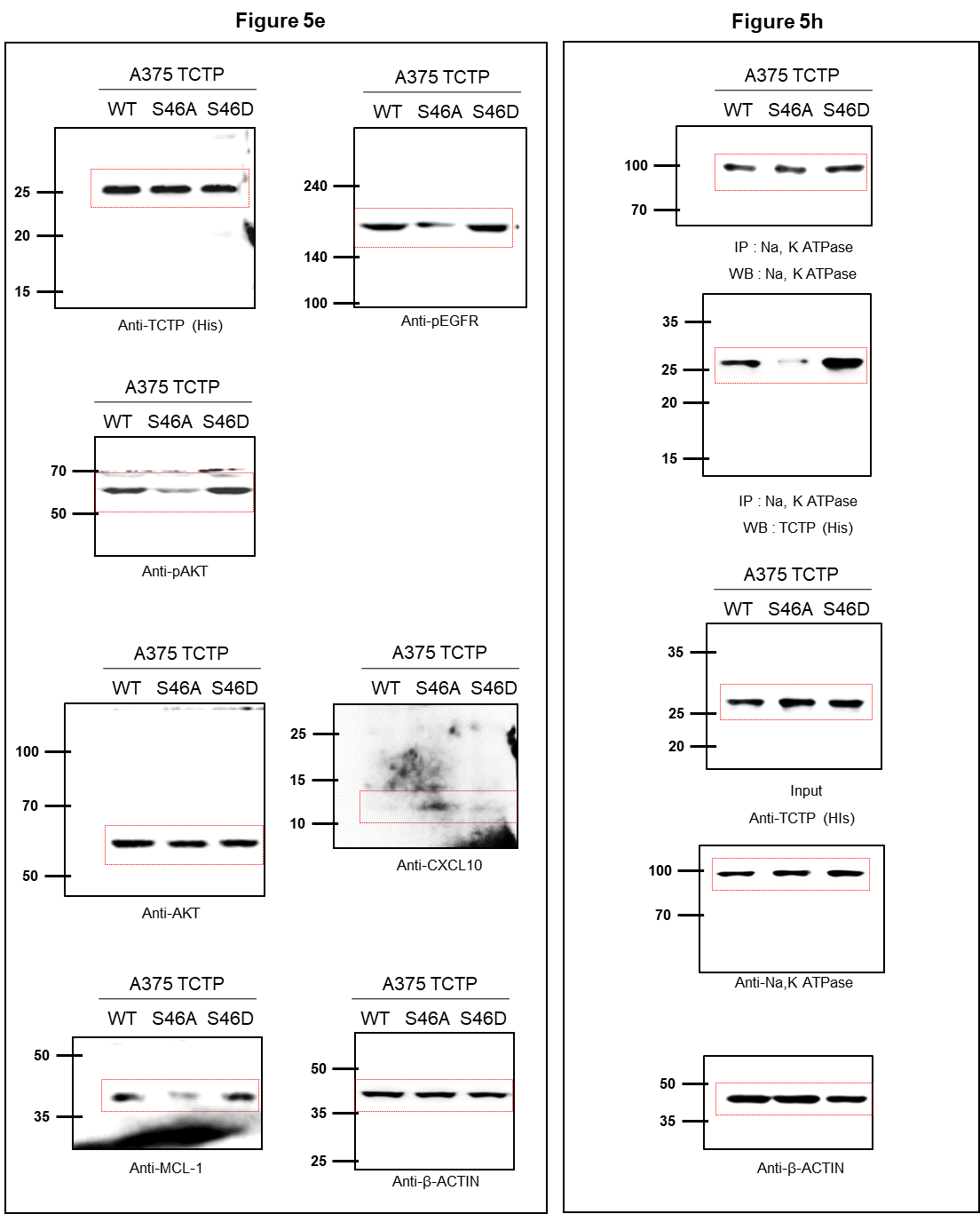


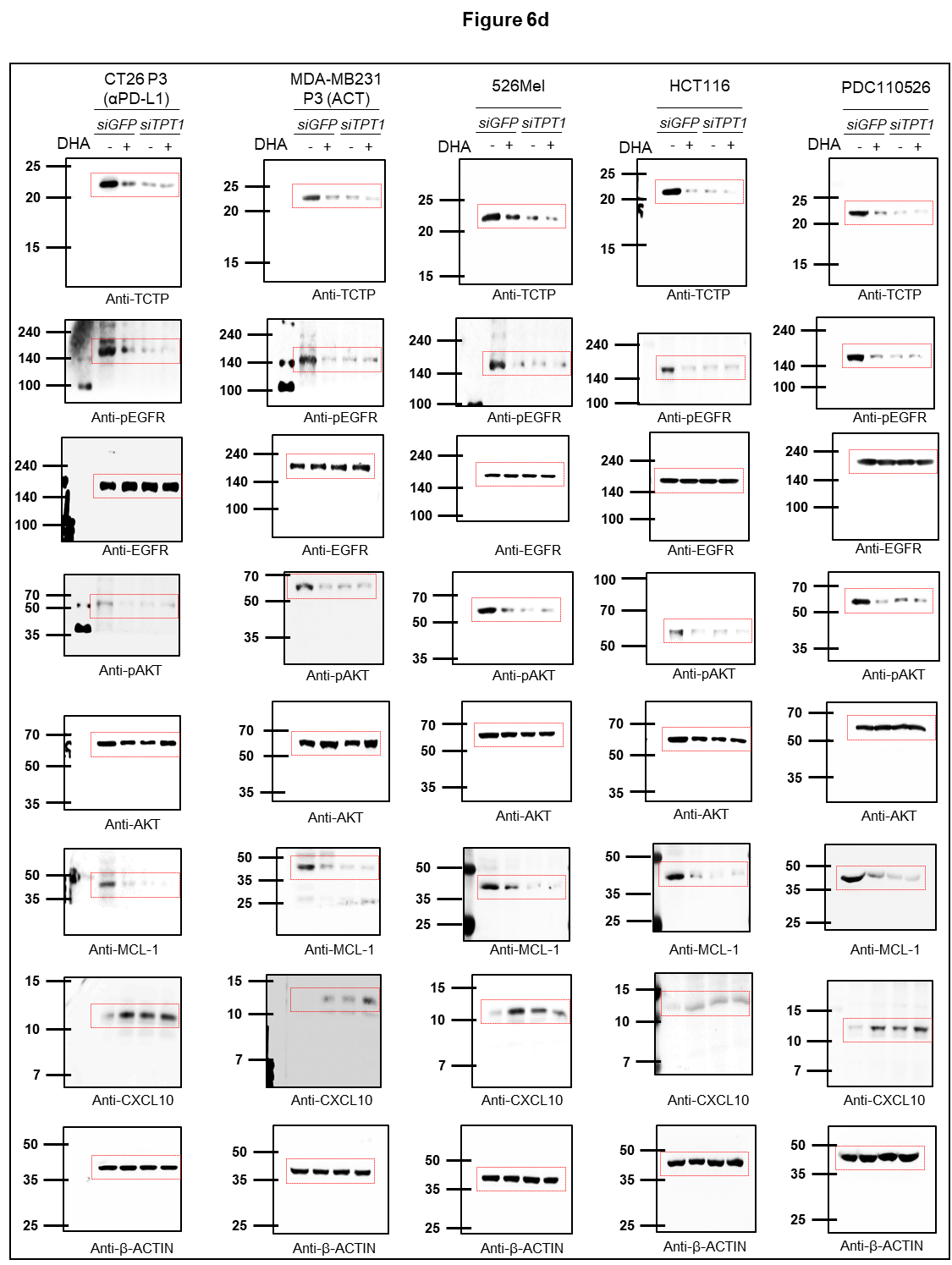


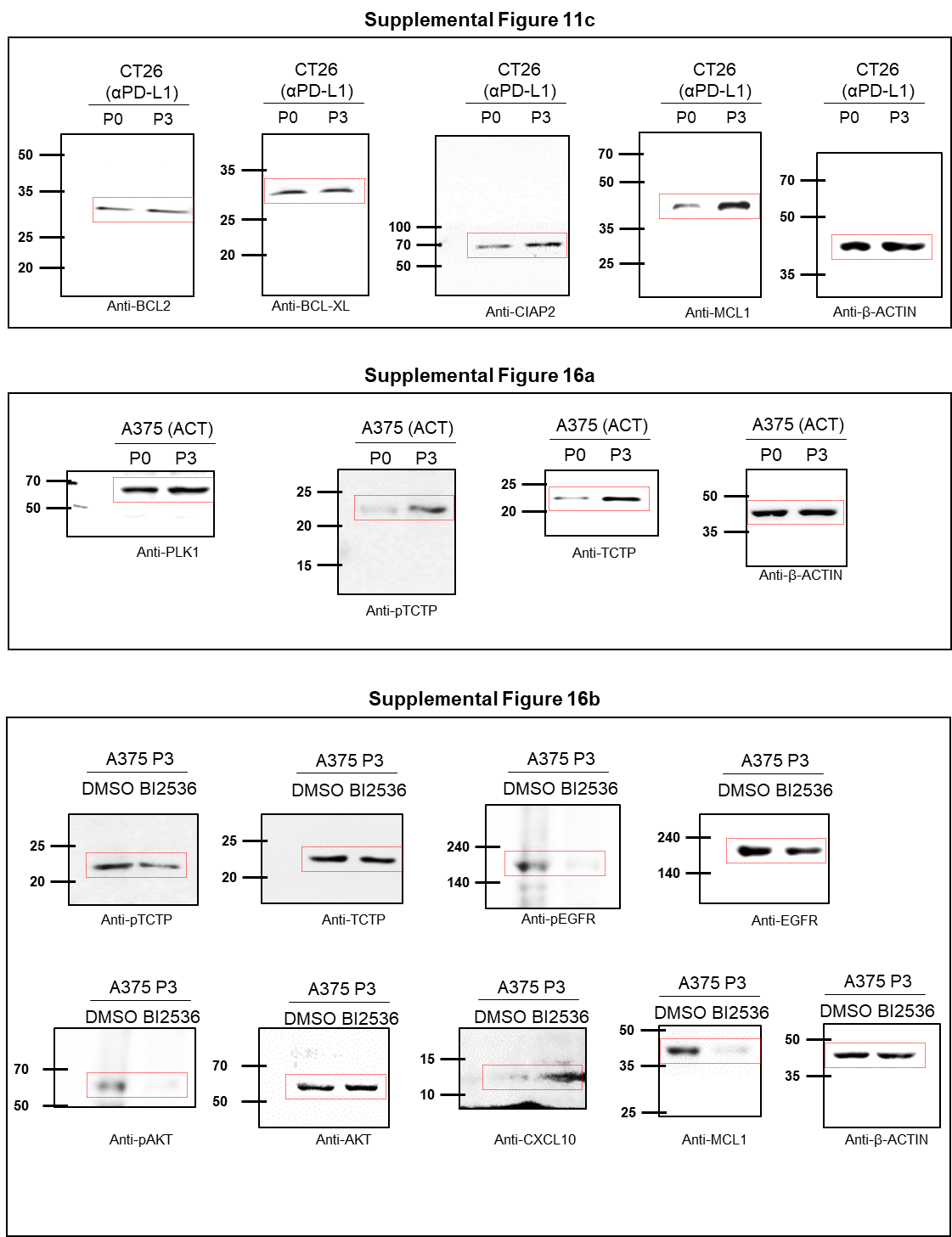


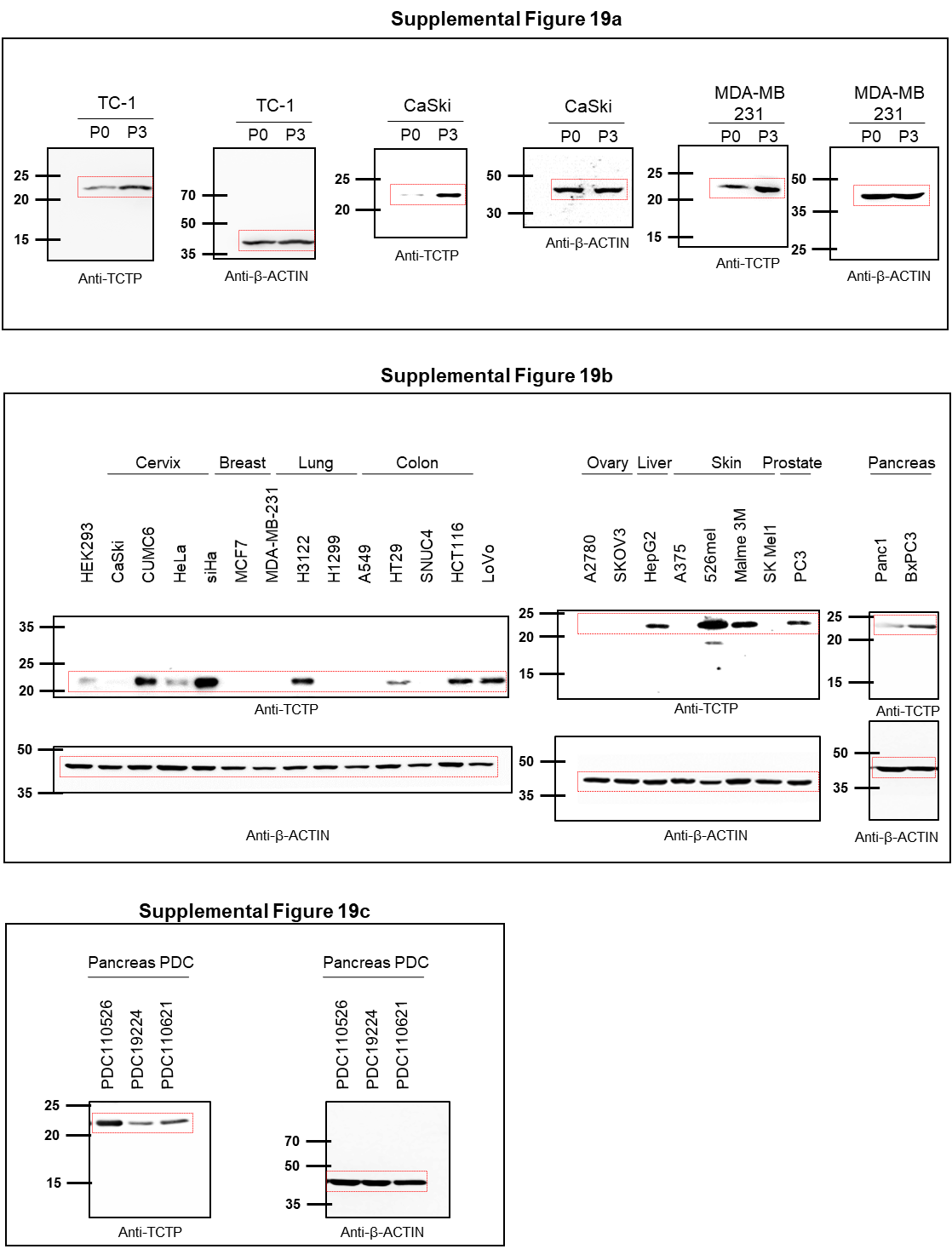


**References**

1. Tavi Nathanson. et al. Somatic Mutations and Neoepitope Homology in Melanomas Treated with CTLA-4 Blockade. *Cancer Immunology research* **5(1)**, 84-91 (2017).

2. David Liu. et al. Integrative molecular and clinical modeling of clinical outcomes to PD1 blockade in patients with metastatic melanoma. *Nature Medicine* **25**, 1916-1927 (2019).

3. Tuba N Gide. et al. Distinct Immune Cell Populations Define Response to Anti-PD-1 Monotherapy and Anti-PD-1/Anti-CTLA-4 Combined Therapy. *Cancer cell* **35(2)**, 238-255 (2019).
