# Supplementary material for: Targeting TCTP sensitizes tumor to T cell-mediated therapy by reversing immune-refractory phenotypes
Source: Nat Commun. 2022 Apr 19;13:2127. doi: 10.1038/s41467-022-29611-y (PMC9019109; doi:10.1038/s41467-022-29611-y)
Supplement: Supplementary file 2 — Reporting Summary [file 41467_2022_29611_MOESM2_ESM.pdf]

nature research

Corresponding author(s): Tae Woo Kim
Last updated by author(s): Feb 17, 2022

# Reporting Summary

Nature Research wishes to improve the reproducibility of the work that we publish. This form provides structure for consistency and transparency in reporting. For further information on Nature Research policies, see our Editorial Policies and the Editorial Policy Checklist.

## Statistics

For all statistical analyses, confirm that the following items are present in the figure legend, table legend, main text, or Methods section.

| n/a | Confirmed | |
|---|---|---|
| ☐ | ☒ | The exact sample size (*n*) for each experimental group/condition, given as a discrete number and unit of measurement |
| ☐ | ☒ | A statement on whether measurements were taken from distinct samples or whether the same sample was measured repeatedly |
| ☐ | ☒ | The statistical test(s) used AND whether they are one- or two-sided<br>*Only common tests should be described solely by name; describe more complex techniques in the Methods section.* |
| ☐ | ☒ | A description of all covariates tested |
| ☐ | ☒ | A description of any assumptions or corrections, such as tests of normality and adjustment for multiple comparisons |
| ☐ | ☒ | A full description of the statistical parameters including central tendency (e.g. means) or other basic estimates (e.g. regression coefficient) AND variation (e.g. standard deviation) or associated estimates of uncertainty (e.g. confidence intervals) |
| ☐ | ☒ | For null hypothesis testing, the test statistic (e.g. *F*, *t*, *r*) with confidence intervals, effect sizes, degrees of freedom and *P* value noted<br>*Give P values as exact values whenever suitable.* |
| ☒ | ☐ | For Bayesian analysis, information on the choice of priors and Markov chain Monte Carlo settings |
| ☒ | ☐ | For hierarchical and complex designs, identification of the appropriate level for tests and full reporting of outcomes |
| ☐ | ☒ | Estimates of effect sizes (e.g. Cohen's *d*, Pearson's *r*), indicating how they were calculated |

*Our web collection on statistics for biologists contains articles on many of the points above.*

## Software and code

Policy information about availability of computer code

| | |
|---|---|
| Data collection | No software was used for data collection. |
| Data analysis | GraphPad Prism Ver. 7.0 (GraphPad Software, Inc., San Deigo, CA, USA), R version 4.0.2, R studio version 1.2.5033, Image J 1.53g, FACSuite software version 1.0.5. |

For manuscripts utilizing custom algorithms or software that are central to the research but not yet described in published literature, software must be made available to editors and reviewers. We strongly encourage code deposition in a community repository (e.g. GitHub). See the Nature Research guidelines for submitting code & software for further information.

## Data

Policy information about availability of data

All manuscripts must include a data availability statement. This statement should provide the following information, where applicable:

- Accession codes, unique identifiers, or web links for publicly available datasets
- A list of figures that have associated raw data
- A description of any restrictions on data availability

RNA sequencing data used in this study have been published (Mariathasan, S. et al. TGFβ attenuates tumour response to PD-L1 blockade by contributing to exclusion of T cells. Nature 554, 544 (2018)) and deposited to European Genome-Phenome Archive under accession number EGAS00001002556 [https://www.omicsdi.org/dataset/ega/EGAS00001002556]. RNA-seq datasets from various cancer types are available in the Cancer Genome Atlas (TCGA) portal (https://tcga-data.nci.nih.gov/tcga/). The gene ontology (GO) analysis which supported the findings of this study is publicly available online at [https://amp.pharm.mssm.edu/Enrichr/]. The source data underlying Figures 1-7 and Supplemental Figures 1-19 are provided in Source data file with this paper. The gating strategy is provided in Supplemental Figure 20. All raw images for the immunoblots are provided in Supplemental Figure 21. All other data supporting the findings of this study are available from the corresponding author on reasonable request.

# Field-specific reporting

Please select the one below that is the best fit for your research. If you are not sure, read the appropriate sections before making your selection.

☒ Life sciences ☐ Behavioural & social sciences ☐ Ecological, evolutionary & environmental sciences

For a reference copy of the document with all sections, see nature.com/documents/nr-reporting-summary-flat.pdf

# Life sciences study design

All studies must disclose on these points even when the disclosure is negative.

| | |
|---|---|
| Sample size | Mice number (n=10) were determined by in vivo experience study to monitor and calculate percentage of mice survival of each experimental group. |
| Data exclusions | No data were excluded. |
| Replication | All data are representative of at least 3 separate experiments. All attempt at replication were successful |
| Randomization | For in vivo treatment experiments, tumor-bearing mice were subjected to caliper measurements. Animals with comparable tumor sizes were randomized into treatment groups; this prevented outcomes from being influenced by initial differences in tumor burden. For in vitro cell experiments, all cells in each experiment were from the same parental cells. |
| Blinding | To give different treatments to different experimental groups, the investigators were not blinded in vivo study. Analyses in the animal experiments as the results reported were based on measurements acquired. For RNA analyses using qRT-PCR and protein analyses by Western blotting were not performed blind to load samples by order. Almost investigators were not blinded because the experiments we performed in this study was not applicable for blinding. |

# Reporting for specific materials, systems and methods

We require information from authors about some types of materials, experimental systems and methods used in many studies. Here, indicate whether each material, system or method listed is relevant to your study. If you are not sure if a list item applies to your research, read the appropriate section before selecting a response.

## Materials & experimental systems

| n/a | Involved in the study | |
|---|---|---|
| ☐ | ☒ | Antibodies |
| ☐ | ☒ | Eukaryotic cell lines |
| ☒ | ☐ | Palaeontology and archaeology |
| ☐ | ☒ | Animals and other organisms |
| ☒ | ☐ | Human research participants |
| ☒ | ☐ | Clinical data |
| ☒ | ☐ | Dual use research of concern |

## Methods

| n/a | Involved in the study | |
|---|---|---|
| ☒ | ☐ | ChIP-seq |
| ☐ | ☒ | Flow cytometry |
| ☒ | ☐ | MRI-based neuroimaging |

## Antibodies

| | |
|---|---|
| Antibodies used | The antibodies used in this study were anti-active caspase-3 antibody(BD Pharmingen, Cat 561011), TCTP (ab37506, Abcam), pTCTP (#5251, Cell signaling), pEGFR(Life technologies, 44784G), EGFR(#4267, Cell signaling), PLK1 (#4535, Cell signaling), MCL1 (sc-819, Santa Cruz Biotechnology), CXCL10 (551215, BD Biosicences), β-actin (M177-3, MBL), anti-rabbit IgG-HRP (Enzo, Cat ADI-SAB-300-J) and anti-mouse IgG-HRP (Enzo, Cat ADI-SAB-100-J). |
| Validation | Validation statements for antibodies can be found on their corresponding manufacturer websites. Validation in our own samples has been confirmed by Western blot detection of bands at the predicted size. Anti-active caspase-3 antibody (BD Pharmingen, Cat 561011) was used for flow cytometry work, and TCTP (ab37506, Abcam), pTCTP (#5251, Cell signaling), pEGFR (Life technologies, 44784G), EGFR (#4267, Cell signaling), PLK1 (#4535, Cell signaling), MCL1 (sc-819, Santa Cruz Biotechnology), CXCL10 (551215, BD Biosicences), β-actin (M177-3, MBL), anti-rabbit IgG-HRP (Enzo, Cat ADI-SAB-00-J) and anti-mouse IgG-HRP (Enzo, Cat ADI-SAB-100-J) were used for western blot analysis. TCTP (ab37506, Abcam) and anti-rabbit IgG-HRP (Enzo, Cat ADI-SAB-300-J) were validated for IHC analysis. |

## Eukaryotic cell lines

Policy information about cell lines

| | |
|---|---|
| Cell line source(s) | A375, CaSki, 526Mel, MDA-MB231, CT26 and HCT116 cell lines were purchased from American Type Culture Collection |

| | |
|---|---|
| | (ATCC, Manassas, VA, USA). 293FT cell line was purchased from Invitrogen. All cell lines were obtained between 2010 and 2014.<br>Details about generation of CT26 P3 and A375 P3 cells lines used in this study are provided in the Materials and Methods section of the manuscript. Generation of the immune edited CaSki P3 cell line is described in Clinical cancer research 2015;21 (6):1438-46. Generation of the immune edited MDA-MB P3 and CaSki-NANOG cell line is described in Cancer research 2017;77(18):5039-53.<br>Pancreas PDC cell lines were generated by Suhwan Chang as described in Cancer Letter 2016;465:82-93. |
| Authentication | The identities of cell lines were confirmed by short tandem repeat (STR) profiling by IDEXX Laboratories Inc. and used within 6 months for testing. |
| Mycoplasma contamination | All cell lines were tested for mycoplasma using Mycoplasma Detection Kit (Thermo Fisher Scientific, San Jose, CA, USA) and negative for mycoplasma contamination. |
| Commonly misidentified lines<br>(See ICLAC register) | No misidentified cell line was used in this study. |

# Animals and other organisms

Policy information about studies involving animals; ARRIVE guidelines recommended for reporting animal research

| | |
|---|---|
| Laboratory animals | 6- to 8- week-old female NOD/SCID or Balb/c mice were used in this project. All mice were maintained in SPF1 (specific pathogen free-1) condition and cared by Korea University Institutional Animal care center policy. |
| Wild animals | This study did not involve wild animals. |
| Field-collected samples | The study did not involve samples collected from the field. |
| Ethics oversight | All mice were maintained and handled under the protocol approved by the Korea University Institutional Animal Care and Use Committee (KOREA-2017-0141). All animal procedures were performed in accordance with recommendations for the proper use and care of laboratory animals. |

Note that full information on the approval of the study protocol must also be provided in the manuscript.

# Flow Cytometry

## Plots

Confirm that:

- [x] The axis labels state the marker and fluorochrome used (e.g. CD4-FITC).
- [x] The axis scales are clearly visible. Include numbers along axes only for bottom left plot of group (a 'group' is an analysis of identical markers).
- [x] All plots are contour plots with outliers or pseudocolor plots.
- [x] A numerical value for number of cells or percentage (with statistics) is provided.

## Methodology

| | |
|---|---|
| Sample preparation | For CTL-mediated apoptosis assay, tumor cells were labeled with CFSE (10 μM, Molecular Probes, Eugene, OR) in DMEM supplemented with 0.1% FBS. The CFSE-labeled tumor cells were pulsed with MART1 peptide (10 μg/ml) for 1 hour if nessesary. The CFSE-labeled tumor cells were mixed with cognate tumor antigen–specific CD8+ CTLs at a 1:1 ratio and incubated for 4 hours at 37°C.<br>For Granzyme B-mediated apoptosis assay, recombinant human granzyme B (Enzo Life Sciences) was mixed with BioPorter Reagent (Sigma-Aldrich) at 25°C for 5 minutes. Tumor cells were mixed with BioPorter–granzyme B complexes for 4-6 hours at 37°C. Cells were stained for active caspase-3 as an index of apoptosis and examined by flow cytometry.<br>For immune cell tumor infiltration, treated mice were sacrificed on day 18 following tumor inoculation and tumors were harvested. Tumors were dissected into fragments by cutting, dissociated by a cell strainer. Cell suspensions were stained for intracellular and extracellular protein markers of interest. |
| Instrument | FACSVerse flowcytometer (BD Biosciences, Cat no. #651154, year 2014) |
| Software | Data analysis was performed in BD FACSuite software. |
| Cell population abundance | Purity was determined by running flow cytometry of the sorted population. |
| Gating strategy | Cell populations were first gated to exclude cell debris and aggregates based on FSC/SSC. Then cells stained with control were used to determine the boundary between "negative" and "positive" cells; this boundary was used to identify positive cells in samples stained with the specific antibody. The gating strategy is provided in Supplementary Figure 20. |

- [x] Tick this box to confirm that a figure exemplifying the gating strategy is provided in the Supplementary Information.
